# AF8c, a Multi-Kinase Inhibitor Induces Apoptosis by Activating DR5/Nrf2 via ROS in Colorectal Cancer Cells

**DOI:** 10.3390/cancers14133043

**Published:** 2022-06-21

**Authors:** Soyeon Jeong, Ahmed K. Farag, Hye Kyeong Yun, Yoon A. Jeong, Dae Yeong Kim, Min Jee Jo, Seong Hye Park, Bo Ram Kim, Jung Lim Kim, Bu Gyeom Kim, Dae-Hee Lee, Eun Joo Roh, Sang Cheul Oh

**Affiliations:** 1 Division of Oncology/Hematology, Department of Internal Medicine, College of Medicine, Korea University, Seoul 08308, Korea; jensyj85@gmail.com (S.J.); ilovewish777@naver.com (B.R.K.); clickkjl@naver.com (J.L.K.); 2 Senior Research Manager, Manufacturing Department, Curachem, Inc., Chungcheongbuk-do 28161, Korea; ahmed@curachem.com or; 3 Graduate School of Medicine, College of Medicine, Korea University, Seoul 08308, Korea; katecoco@hanmail.net (H.K.Y.); leomi2614@naver.com (Y.A.J.); derrickdyblue22@gmail.com (D.Y.K.); minjeeyoyo@nate.com (M.J.J.); psh3938@hanmail.net (S.H.P.); qnrua10047@naver.com (B.G.K.); 4 Department of Marine Food Science and Technology, Gangneung-Wonju National University, Gangwon 210-702, Korea; 5 Chemical Kinomics Research Center, Korea Institute of Science and Technology (KIST), Seoul 02792, Korea; 6 Division of Bio-Medical Science &Technology, KIST School, University of Science and Technology, Seoul 02792, Korea

**Keywords:** AF8c, colorectal cancer (CRC), apoptosis, death receptors (DRs), ER stress, reactive oxygen species (ROS), nuclear respiratory factor 2 alpha subunit (Nrf2), kinase, polypharmacological molecules

## Abstract

**Simple Summary:**

AF8c, a lapatinib hybrid quinazoline-based EGFR/HER2 inhibitor, was chosen to scrutinize its antiproliferative activity in colorectal cancer (CRC) cells. We found that AF8cinduced apoptosis in CRC cells via diverse mechanisms. In addition to inhibiting the phosphorylation of the ErbB family, AF8c increased the mRNA and protein levels of death receptor 5 (DR5) in vitro and in vivo. In addition, AF8c upregulated several ER stress proteins and the redox-sensitive nuclear respiratory factor 2 alpha subunit (Nrf2) in a p53-dependent manner. We also found that the AF8c-induced increase in the levels of Nrf2, DR5, and apoptosis was diminished by p53 downregulation or knockdown. Furthermore, AF8c showed higher antiproliferative activity than lapatinib in the CRC mouse model in vivo. Therefore, our results suggest AF8c as a highly effective polypharmacological small molecule with an encouraging safety profile, both in vitro and in vivo, for further evaluation as a treatment of CRC.

**Abstract:**

Our team has previously reported a series of quinazoline-based lapatinib hybrids as potent kinase-targeting anticancer agents. Among them, AF8c showed a relatively safe profile in colorectal cancer (CRC) cells. In this study, we delineate a novel anticancer activity of AF8c in CRC cells. AF8c mediated p53-dependent apoptosis of CRC cells via the generation of endoplasmic reticulum (ER) stress and reactive oxygen species (ROS), as well as activation of nuclear respiratory factor 2 alpha subunit (Nrf2) and death receptor 5 (DR5), among others. The silencing of DR5 attenuated the expression levels of Nrf2 and partially inhibited AF8c-induced apoptosis. Additionally, upregulation of Nrf2 by AF8c evoked apoptosis through a decrease in antioxidant levels. Treatment of a CRC mice model with AF8c also resulted in the upregulation of DR5, Nrf2, and CHOP proteins, subsequently leading to a significant decrease in tumor burden. In comparison with lapatinib, AF8c showed higher cellular antiproliferative activity at the tested concentrations in CRC cells and synergized TRAIL effects in CRC cells. Overall, our results suggest that AF8c-induced apoptosis may be associated with DR5/Nrf2 activation through ER stress and ROS generation in CRC cells. These findings indicate that AF8c represents a promising polypharmacological molecule for the treatment of human CRC.

## 1. Introduction

Colorectal cancer (CRC) has been gaining considerable attention, being the third most commonly diagnosed and the second most mortality-causing cancer in men and women in the US [1]. This high mortality rate is attributed, at least in part, to poor prognosis and the rapid metastasis rate, which occurs in 35% of patients at the diagnosis stage and ultimately occurs in 50% of patients diagnosed with non-metastasizing CRC [2]. The standard therapeutic protocol in CRC is surgery and chemo/radiotherapy [3]. Targeted therapy, including bevacizumab, cetuximab, and ziv-aflibercept, however, is gaining considerable momentum as they avoid the shortcomings of typical chemotherapy [4]. Despite advances in surgery, chemotherapeutics, and targeted therapy to treat CRC, the overall clinical efficacy of these agents remains limited due to disease heterogeneity and the rapid resistance development [5,6,7]. Therefore, there is an urgent need for more effective strategies to treat CRC patients.

Triggering apoptosis is one of the effective strategies for targeting cancers. In multicellular organisms, apoptosis is the innocuous process of discarding unneeded cells through intrinsic and extrinsic pathways, both of which ultimately activate the effector caspases. The intrinsic mitochondrial pathway is initiated within the cell in response to intracellular factors such as oxidative stress. Stimulation of the intrinsic pathway is the result of the release of cytochrome c and mitochondrial membrane permeability [8]. On the other hand, the extrinsic pathway is triggered by the activation of cell surface receptors known as death receptors (DRs) [9]. The DRs belong to the tumor necrosis factor (TNF) receptor superfamily that trimerize upon ligand binding, leading to the recruitment of an adapter protein to the cytoplasmic death domain. Among the DRs, DR4 and DR5 are the functional cell surface receptors that mediate TNF-related apoptosis-inducing ligand (TRAIL)-induced apoptosis. The merits of targeting the TRAIL pathway for cancer therapy have been thoroughly discussed in the literature, as it selectively induces apoptosis in cancer cells, sparing normal cells [10,11,12,13,14,15].

The intracellular level of reactive oxygen species (ROS) is a critical factor in determining the fate of the cell [16,17]. While low to intermediate levels of ROS are required by most cells to maintain their normal physiological functions, such as cell signaling and protein phosphorylation, high levels of ROS are associated with the development and progression of cancer. Prolonged elevation of localized ROS causes cell death. ROS are produced as byproducts of oxidative phosphorylation in the mitochondria [18]. Physiologically, ROS are cleared by diverse mechanisms via enzymatic and non-enzymatic mechanisms. Failure to detoxify the generated ROS results in their accumulation inside the cells, non-specific damage to proteins and other cellular components, and ultimately cell death. Several anticancer agents have been found to elicit their antiproliferative effects via stimulating the overproduction of ROS and/or disrupting their clearance mechanisms [19].

**Figure 1:**
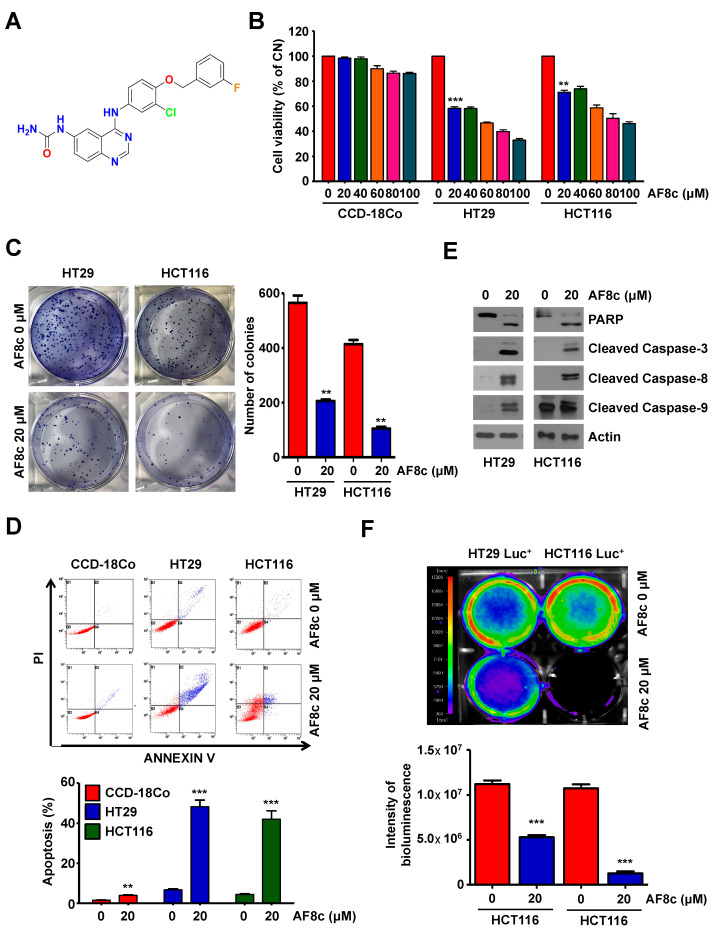
AF8c induces apoptotic cell death in human CRC cells: (**A**) the chemical structure of AF8c; (**B**) normal colon CCD-18Co (**left**), CRC HT29 (**middle**), and HCT116 (**right**) cells were treated with 0–100 μM AF8c for 24 h, and cell viability was then assessed using the WST-1 assay. **, *p* < 0.01 and ***, *p* < 0.001; (**C**) after treatment with DMSO or 20 μM AF8c, cells were stained with crystal violet and imaged (**left**); the graph (**right**) is a quantification of colony formation. **, *p* < 0.01; (**D**) Cells were incubated with 20 μM AF8c for 24 h and then analyzed using flow cytometry after staining with Annexin V and PI; (**E**) HT29 (**left**) and HCT116 (**right**) cells were treated with AF8c for 24 h, and the expression levels of apoptosis-associated proteins were determined by immunoblotting; confirmation experiments: HT29 (**left**) and HCT116 (**right**) cells were pretreated with Z-VAD-fmk for 1 h, then treated with AF8c for 24 h, and the caspase cascade was confirmed by Western blot; (**F**) HT29 Luc^+^ (**left**) and HCT116 Luc^+^ (**right**) cells were plated in 6-well plates and then treated with 20 μM AF8c. Images were obtained using the charge-coupled camera of the in vivo imaging system (**upper**) and quantified (**lower**). ***, *p* < 0.001.

A number of studies reported novel antiproliferative mechanisms of action (MOAs) for FDA-approved anticancer drugs [20,21,22]. These novel MOAs, which are considered an off-target activity, significantly contributed to the observed potency of these drugs. Lapatinib, the FDA-approved epidermal growth factor receptor (EGFR) and human epidermal growth factor receptor 2 (HER2) inhibitor, is an example as it was found to improve the apoptotic effect of TRAIL and TRAIL-Rs agonists, such as mapatumumab and lexatumumab in CRC cells via upregulation of DR4 and 5 [23]. In addition to lapatinib, the benefits of the synergistic combination of TRAIL therapy with EGFR/HER2 inhibition in overcoming TRAIL therapy resistance and improving the therapeutic outcomes were suggested. Trastuzumab, the FDA-approved HER2 antibody, was found to downregulate HER2 (ErbB2) receptors and enhance TRAIL-mediated apoptotic effects in ovarian and breast cancer cell lines [24]. The effect of TRAIL alone on these cells was limited in comparison with the trastuzumab/TRAIL combination [25]. Furthermore, EGFR (ErbB1) activation was significantly correlated to TRAIL-therapy resistance. Gefitinib, the FDA-approved EGFR inhibitor, was reported to sensitize human bladder cancer cell lines to TRAIL therapy via inhibition of protein kinase B (Akt) and X-linked inhibitor of apoptosis protein (XIAP) cascades [26]. In light of these reports, we tested the cellular activity of our reported series of lapatinib-derived dual EGFR/HER2 inhibitors in HT29 and HCT116 CRC cells [27]. Within this series, we selected the compound we named AF8c (Figure 1A) for further scrutinization of its mode of action in CRC cells as it preferentially induced cytotoxicity in CRC cells without affecting CCD-18CO normal epithelial primary colon cells, thus representing an interesting compound for further development against CRC.

## 2. Materials and Methods

### 2.1. Cells and Cell Culture

Human CRC HT29, HCT116, and CCD-18CO (normal colon) cell lines were purchased from American Type Cell Culture Collection. HT29 Luc^+^ and HCT116 Luc^+^ cells were obtained from the JCRB Cell Bank. HT29, HCT116, HT29 Luc^+^, and HCT116 Luc^+^ cells were maintained in McCoy’s 5A medium, while CCD-18Co cells were maintained in Eagle’s Minimum Essential Medium. The media were supplemented with 10% heat-inactivated fetal bovine serum (FBS), penicillin, and streptomycin. The cells were cultured in a humidified atmosphere with 5% CO_2_ at 37 °C.

### 2.2. Chemicals and Antibodies

Synthesis of AF8c was performed as previously reported and was purified to obtain an HPLC purity of greater than 99.9% [27]. AF8c and MitoSOX Red were dissolved in dimethyl sulfoxide (DMSO), while N-acetyl-L-cysteine (NAC) was dissolved in phosphate-buffered saline (PBS). Hydrogen peroxide (H_2_O_2_) was dissolved in distilled water and was stored at −20 °C. The cells were incubated with 1 mM NAC for 1 h before treatment with either AF8c or H_2_O_2_ and were incubated for 24 h before being harvested. caspase inhibitor Z-VAD-fmk was purchased from Promega. The cells were treated with Z-VAD-fmk for 1 h before incubation with AF8c.

The antibodies used and their sources are as follows: Cleaved Poly (ADP-ribose) polymerase (PARP), caspase-3, caspase-9, Cleaved caspase-8, SOD1, SOD2, Catalase, nuclear factor erythroid 2–related factor 2 (Nrf2), Kelch-like ECH-associated protein 1 (Keap1), inositol-requiring enzyme-1α (IRE1α), phospho-IRE1α, activating transcription factor 6 (ATF6), PKR-like ER-resistant kinase (PERK), phospho-PERK, Eukaryotic translation initiation factor 2A (eIF2α), phospho-eIF2α, Glucose Regulated Protein 94 (GRP94), ATF, Bip, ErbB1, ErbB3, ErbB4, and phospho-ErbB1 antibodies were purchased from Cell Signaling Technology. The antibodies against SOD3, C/EBP homologous protein (CHOP), DR4, Nrf2, Lamin B, Ubi, ErbB2, phospho-ErbB1, phospho-ErbB2, phospho-ErbB3, and phospho-ErbB4 were purchased from Santa Cruz. The antibodies against β-Actin were purchased from Sigma Aldrich. DR5 was obtained from the R&D systems (MN, USA); goat anti-rabbit and goat anti-mouse secondary antibodies were purchased from Cell Signaling Technology (MA, USA).

### 2.3. Cell Viability Assay

Cell viability was examined using the Ez-Cytox cell viability assay kit (DoGen, Seoul, South Korea). The cells were seeded into 96-well plates (1 × 10^4^ cells per well) and incubated for 1 day at 37 °C. The cells were then treated with AF8c for 24 h. The cells were incubated with Ez-Cytox for 2 h, and the absorbance was measured with a spectrophotometer at 450 nm.

### 2.4. Colony Formation

The cells were plated on 60-mm dishes and treated with 20 μM AF8c for 24 h. The cells were then detached by trypsinization and seeded in 6-well plates. After 2 weeks, the cells were stained with crystal violet.

### 2.5. Western Blot Analysis

Western blot analysis was performed as previously described [28]. Original Western Blot images can be found at Figure S6.

### 2.6. Analysis of Apoptosis

Apoptosis was measured using an Annexin V-Fluorescein Isothiocyanate (FITC) Apoptosis Detection Kit (BioBud, Seoul, Korea, Cat. LS-02-100). The cells were either treated or not treated with AF8c and harvested. The harvested cells were stained by mixing Annexin V-FITC and propidium iodide (PI) reagent with 1× binding buffer for 30 min at room temperature in the dark and analyzed by flow cytometry.

### 2.7. Determination of Mitochondrial ROS

The generation of ROS was determined by flow cytometry after staining with dihydroethidium (DHE) and MitoSOX to specifically detect the generation of intracellular and mitochondrial superoxide species. Cells were plated on a coverslip or in a 6-well plate and incubated with or without 20 μM AF8c for 4 h. For cells stained with 10 μM DHE or MitoSOX, fluorescent dyes were added to the cell media, and the cells were incubated at 37 °C for 30 min. For each sample, at least 10,000 events were acquired and analyzed using a Beckman Coulter Navios flow cytometer (Beckman Coulter Life Sciences Inc., Brea, CA, USA). Alternatively, MitoSox-stained cells were visualized under a confocal microscope.

### 2.8. Transfection

Small interfering RNAs (siRNAs) for the negative control (siNC), DR5 (siDR5), Nrf2 (siNrf2), Keap1 (siKeap1), CHOP (siCHOP), ErbB1 (siErbB1), ErbB2 (siErbB2), ErbB3 (siErbB3), and ErbB4 (siErbB4) were purchased from Santa Cruz Biotechnology (Santa Cruz, CA, USA). For transfection, 25 nM siRNA was added to 9 × 10^5^ cells in a 60 mm dish using Lipofectamine RNAiMAX, according to the manufacturer’s instructions.

### 2.9. RNA Isolation, and Quantitative Real-Time PCR (qRT-PCR)

Total RNA was isolated using TRIzol reagent (Life Technologies, Carlsbad, CA, USA), and cDNA was synthesized from 2 μg of total RNA. The cDNA was used for qRT-PCR using the TaqMan gene expression master mix reaction system (Applied Biosystems, CA, USA). Gene expression levels were normalized to the level of GAPDH expression.

### 2.10. Immunoprecipitation (IP)

Lysates were incubated with 300 μL Lysis buffer (1 mM PMSF, protease inhibitors, and phosphatase inhibitors) and analyzed for bicinchoninic acid by centrifugation at 15,000 rpm for 5 min at 4 °C for quantification. The supernatant was incubated overnight with primary antibody at 4 °C, and Protein G PLUS-Agarose beads were added at 4 °C for 1 h. Immunoprecipitants were washed and isolated by centrifugation at 15,000 rpm and heated with 2× sample buffer. Then, the supernatant was evaluated by Western blot.

### 2.11. Fraction

For cytoplasm and nuclear fractions, NE-PERTM Nuclear and Cytoplasmic Extraction Reagents were used. The harvested cells were washed with trypsin-EDTA and centrifuged at 500× *g* for 5 min. Cold CER I was added to the pellet, vortex, and incubated on ice for 10 min. CER II vortex was added briefly, and centrifuged to transfer the supernatant (cytoplasm in extract) to a new EP tube, NER was put in the tube with the pellet and placed on ice for 40 min while vortexing every 10 min. Centrifuged for 10 min and then the supernatant (Nuclear extract) was transferred to a new tube. After quantification, Western blot was performed.

### 2.12. Immunofluorescence Staining

After treatment, CRC cells were fixed, blocked, and stained with DR5 or Nrf2 (1:200) primary antibodies, visualized using an anti-rabbit or anti-mouse IgG conjugated to Alexa Fluor-488 or -594, and counterstained with 4′,6-diamidino-2-phenylindole (DAPI) diluted 1:1000. These fluorescently labeled cells were then observed by confocal microscopy.

### 2.13. 3D Cell Culture

Cells (2 × 10^4^ cells per well) were seeded into a 96-well plate for 3D cell culture and incubated for 2 days. After centrifuging the plate, the cells were treated with AF8c and stained with caspase-3/-7 dye for 30 min. The cells were then analyzed using the IncuCyte Live-Cell Analysis System while incubating in the IncuCyte ZOOM, taking pictures every 3 h.

### 2.14. In Vivo Tumor Xenograft Study

The 4-week-old female BALB/c nude mice were injected subcutaneously with either HT29 Luc^+^ or HCT116 Luc^+^ cells (1 × 10^7^ cells in 100 μL PBS). When the tumor had reached approximately 100 mm^3^ in size, the mice were randomly divided into three groups (*n* = 8): DMSO-treated, treated with 10 mg/kg AF8c, and treated with 20 mg/kg AF8c. The tumor size was measured twice a week using calipers. The tumor size was calculated as length × width, while the volume was calculated as 0.5 × length × (width)^2^.

### 2.15. Immunohistochemistry

Tumor cell tissues were incubated with anti-DR5 and anti-Nrf2 primary antibodies and then counterstained with DAPI. Stained tumor tissues were observed under a confocal microscope using ZEN software (version 1.1.13064.302).

### 2.16. Terminal Deoxyribonucleotidyl Transferase-Mediated Deoxyuridine Triphosphate Nick-End Labeling (TUNEL) Assay

The implanted tumor tissues were stained using an in situ cell death detection kit. The staining was performed as per the manufacturer’s instructions. The paraffin tissue section slide was deparaffinized with Xylene and rehydrated while lowering the concentration of EtOH. After washing the slides with PBS, Proteinase K solution was added to the slides and incubated for 10 min. After fixation and permeabilization of the slide, the rTdT Incubation Buffer in the kit was added to the tissue, and then covered with a coverslip and incubated at 37 °C for 1 h. Then, the slides were washed, Dapi stained, dehydrated, and mounted.

### 2.17. Patient-Derived Colorectal Cancer (PDC) Cells

The Institutional Review Board of Guro Hospital (KUGH16275) approved the receipt of tissue donations from CRC patients by the Korea University Guro Hospital tissue bank.

### 2.18. Statistical Analysis

Each experiment was repeated independently at least three times. Statistical analyses were conducted using GraphPad InStat 6.0 software. Data were analyzed using unpaired Student’s *t*-tests. In all analyses, the level of statistical significance was set to a 95% confidence level (*p* < 0.05). *, **, and *** indicate *p* < 0.05, *p* < 0.01, and *p* < 0.001, respectively.

## 3. Results

### 3.1. Treatment with AF8c Inhibits Cell Viability and Induces Apoptosis in Human CRC Cells

In our previous report, we concluded that AF8c inhibits EGFR/Her2 kinases in an in vitro enzymatic assay. However, in this study, we decided to explore the ability of AF8c, as a kinase inhibitor, to inhibit the phosphorylation of the ERBb family members in the cellular context. The results showed that AF8c inhibits the phosphorylation of all members of the ErbB family in both HT29 and HCT116 CRC cells (Figure S1A,B). In a cell-free assay, AF8c inhibited several EGFR mutant forms (Figure S1C), which could make AF8c less prone to immediate resistance.

To investigate the effect of AF8c on the viability of human CRC cells, a range of AF8c concentrations (0–100 μM) were assayed using the WST-1 assay in normal primary colon CCD-18Co cells and two CRC cell lines (HT29 and HCT116) for 24 h. AF8c attenuated cell viability in a dose-dependent manner in CRC cells but not in normal epithelial primary colon CCD-18Co cells (Figure 1B). Additionally, a colony formation assay was performed to examine clonogenic survival in AF8c-treated cells. The colony-forming ability of both CRC cell lines was reduced following AF8c exposure (Figure 1C).

To understand whether the reduced viability observed in the CRC cells after AF8c treatment was attributable to increased apoptosis, we detected the number of Annexin V/PI double-stained cells using flow cytometry. AF8c treatment led to an elevation in the number of Annexin V/PI double-stained cells (Figure 1D) and a significant increase in the levels of cleaved PARP, caspase-3, -8, and -9 (Figure 1E), the well-known apoptotic indicators. To confirm the obtained results, HT29 and HCT116 cells were pretreated with Z-VAD-fmk, the pan-caspase inhibitor, for 30 min. Pretreatment of HT29 and HCT116 cells with Z-VAD-fmk reduced the AF8c-induced cleaved PARP and caspases activities (Figure 1E). The effects of AF8c on the cell viability were then confirmed using in vivo bioluminescence imaging. AF8c treatment led to reduced bioluminescence intensities in HT29 and HCT116 cells (Figure 1F). Collectively, the obtained results suggest that AF8c significantly induces apoptosis in human CRC cells.

As a lapatinib derivate, we decided to compare the activities of AF8c and lapatinib in CRC cells. We found that AF8c attenuated cell viability and promoted apoptosis (as measured by cleaved PARP) to a greater extent than lapatinib at all the tested concentrations, in both cell lines, providing indirect evidence that AF8c could be more effective than lapatinib for CRC therapy (Figure S2A,B). Similar to lapatinib, AF8c sensitized the tested CRC cells to TRAIL treatment (Figure S2C). Additionally, AF8c-triggered apoptotic effects were not abolished by silencing ErbB family members (Figure S1D,E). Taken together, we concluded that the cytotoxic effects of AF8c in CRC cells were stronger than those of lapatinib, and those effects of AF8c were independent of its ErbB kinase family inhibition.

### 3.2. AF8c-Mediated Upregulation of DR5 Is a Factor for Apoptosis Induction

**Figure 2:**
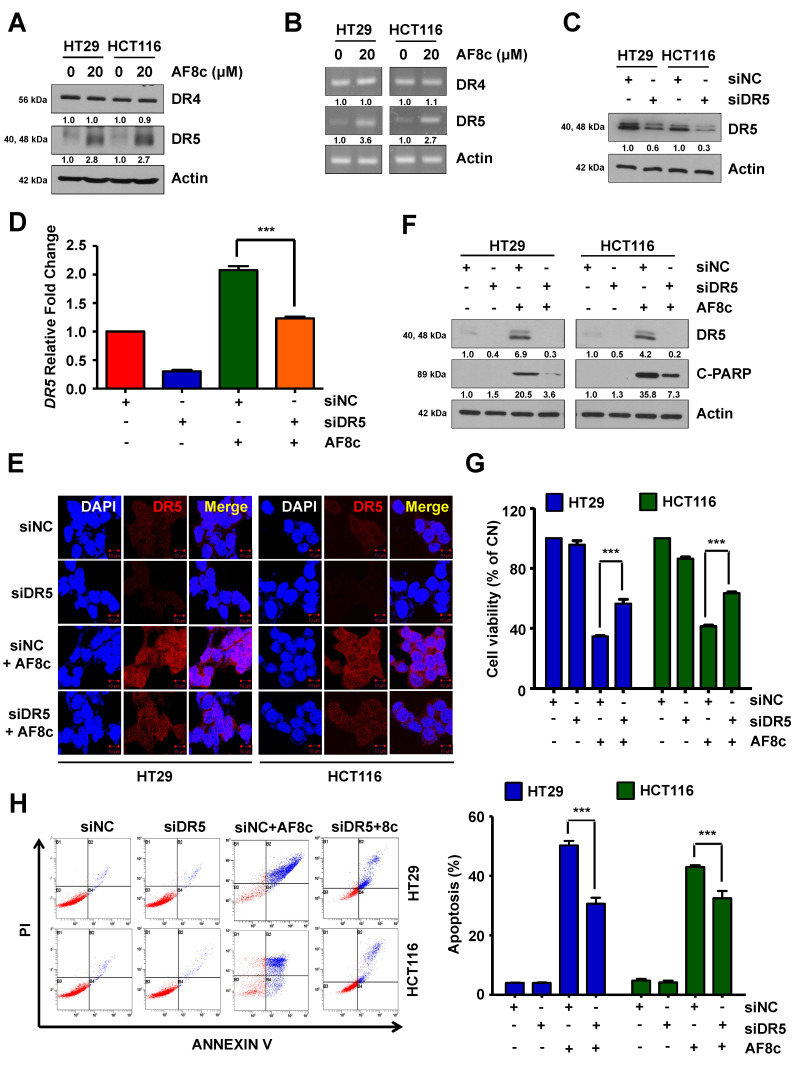
DR5 activation mediates AF8c-induced apoptosis: (**A**) the cells were incubated with AF8c for 24 h and subjected to immunoblotting using antibodies against DR4 and DR5; (**B**) *DR4* and *DR5* mRNA expression levels were analyzed by qRT-PCR; (**C**) after transfection with siRNAs for the negative control (siNC) or DR5 (siDR5), HT29 (**left**) and HCT116 (**right**) cells were analyzed using immunoblotting using antibodies against DR5 and actin; (**D**–**H**) after transfection with siNC or siDR5, cells were treated with or without 20 μM AF8c. mRNA levels (**D**) and fluorescence intensity (**E**) were determined using qRT-PCR and immunofluorescence (scale bars, 10 μm). Cell viability and apoptosis were assayed by immunoblotting (**F**), WST-1 analysis (**G**), and flow cytometry (**H**). ***, *p* < 0.001.

As lapatinib treatment to CRC cells induced the expression of DR4/5, we decided to investigate whether AF8c was able to upregulate DR4/5 similar to lapatinib. In addition, we decided to investigate the role of DR4/5 in AF8c-induced apoptosis. We measured the expression levels of DR4 and DR5 in AF8c-treated cells. The results showed that, while AF8c treatment resulted in increased DR5 expression at both the mRNA and protein levels, surprisingly, no changes were observed in DR4 mRNA or protein expression levels (Figure 2A,B).

To explore the relationship between AF8c-induced apoptosis and DR5 activation, we used siRNA to silence the DR5 gene and treated the CRC cells with AF8c (Figure 2C). Knockdown of DR5 partially restored the levels of DR5 mRNA that were upregulated by AF8c (Figure 2D,E). Additionally, we found that the AF8c-induced apoptosis was significantly suppressed by DR5 knockdown as measured by the levels of cleaved PARP, the viability percentage of the CRC cells, and the percentage of cells undergoing apoptosis (Figure 2F–H). Together, these results suggest that AF8c triggers DR5 activation, which is one of the reasons behind the observed apoptosis in CRC cells.

### 3.3. ER Stress and ROS Leads to Apoptosis in Cells Exposed to AF8c

**Figure 3:**
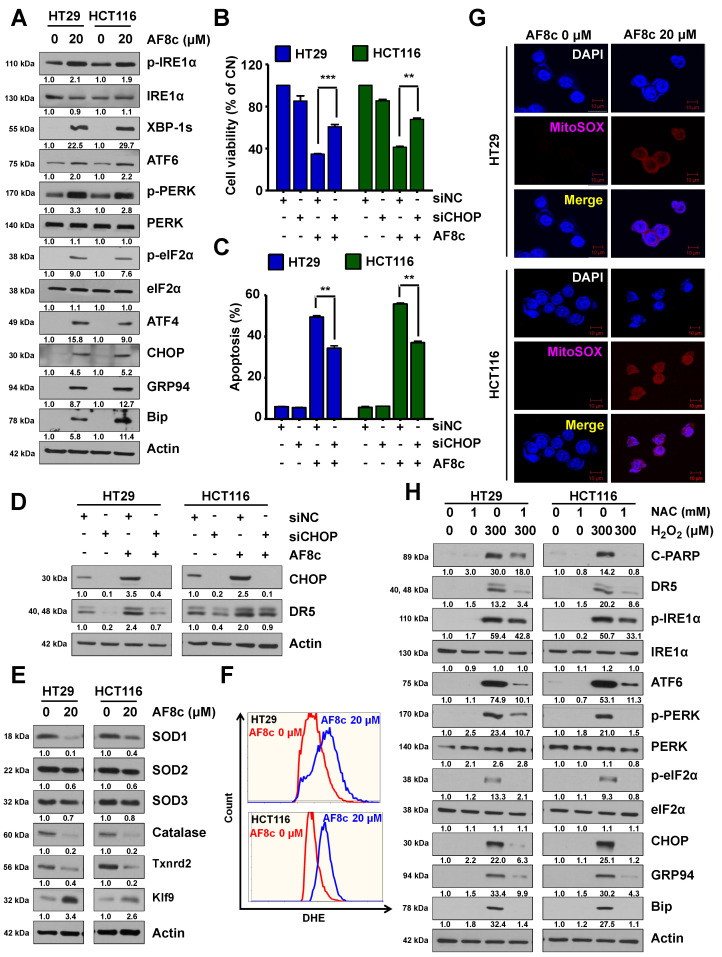
Excessive ROS and ER stress generation by AF8c treatment contribute to DR5-mediated apoptosis: (**A**) HT29 (**left**) and HCT116 (**right**) cells were exposed to 20 μM AF8c for 24 h, and the expression levels of ER stress-related proteins were examined by Western blot; (**B**–**D**) the indicated CRC cells were treated with siNC or siRNAs for CHOP (siCHOP) for 18 h. The cells treated with 20 μM AF8c were then analyzed using WST-1 assay (**B**); FACS analysis (**C**); and Western blotting (**D**). **, *p* < 0.01 and ***, *p* < 0.001. (**E**) After incubating HT29 (**upper**) and HCT116 (**lower**) cells with AF8c, ROS levels were measured using immunofluorescence; (**F**,**G**) the AF8c-treated HT29 (**upper**) and HCT116 (**lower**) cells were stained with 10 μM DHE (F); and MitoSOX (**G**). The cells were then observed by flow cytometry and confocal microscopy (Scale bar, 10 μm). (**H**) HT29 (**left**) and HCT116 (**right**) cells were treated with 300 μM H_2_O_2_ for 24 h, and the expression levels of Cleaved PARP, DR5, and ER stress-related proteins were analyzed by immunoblotting.

In an attempt to understand the reason why AF8c would upregulate DR5, we assumed that ER stress-related proteins such as CHOP could be involved. CHOP protein is one of the major ER stress proteins that regulate the expression of DR5 protein levels [29]. Thus, we investigated whether the observed AF8c-induced DR5 activation was related to ER stress by evaluating the expression levels of ER stress-related proteins in AF8c-treated CRC cells. AF8c significantly augmented the levels of ER stress-related proteins (Figure 3A), such as CHOP, GRP94, and ATF4. Furthermore, AF8c (20 µM) increased the phosphorylation of IRE1α, elF2α, and PERK. CHOP protein knockdown reduced apoptotic cell death and the AF8c-induced increase in DR5 protein levels (Figure 3B–D). These results indicated that the observed apoptotic effects of AF8c in CRCs could be related to AF8c modulating ER stress proteins.

Because ROS levels have been linked to ER stress [30], we tested whether AF8c could induce ROS generation in CRC cells. We found that AF8c affected antioxidants and intracellular ROS levels. While the levels of SOD3 and catalase were decreased by AF8c treatment, the expression of Klf9, a key oxidative stress inducer, was increased, and the expression of its downstream target Txnrd2 was reduced (Figure 3E). Moreover, ROS generation was confirmed in AF8c-treated cells using the dihydroethidium (DHE) experiment (Figure 3F). Next, to determine whether the increased levels of intracellular ROS induced by AF8c exposure originated from mitochondria, the main site of intracellular ROS production, we used the fluorescent probe MitoSOX to stain mitochondrial superoxide. The results showed a higher MitoSOX staining intensity in AF8c-treated cells than in control cells (Figure 3G) in both CRC cell lines.

To confirm by a different approach that the increased ER stress in AF8c-treated CRC cells resulted from excessive ROS generation, HT29 and HCT116 cells were exogenously treated with hydrogen peroxide (H_2_O_2_). Exposure to H_2_O_2_ increased the levels of DR5 and ER stress-related proteins and cleaved PARP in a similar manner to what AF8c does. The antioxidant NAC effectively abolished this effect in both CRC cell lines (Figure 3H). This implies that ROS and ER stress accumulation is required for DR5 activation, as well as for apoptosis, in CRC cells.

### 3.4. Activation of DR5-Induced Nrf2 by AF8c Is a Prerequisite for Apoptotic Cell Death

**Figure 4:**
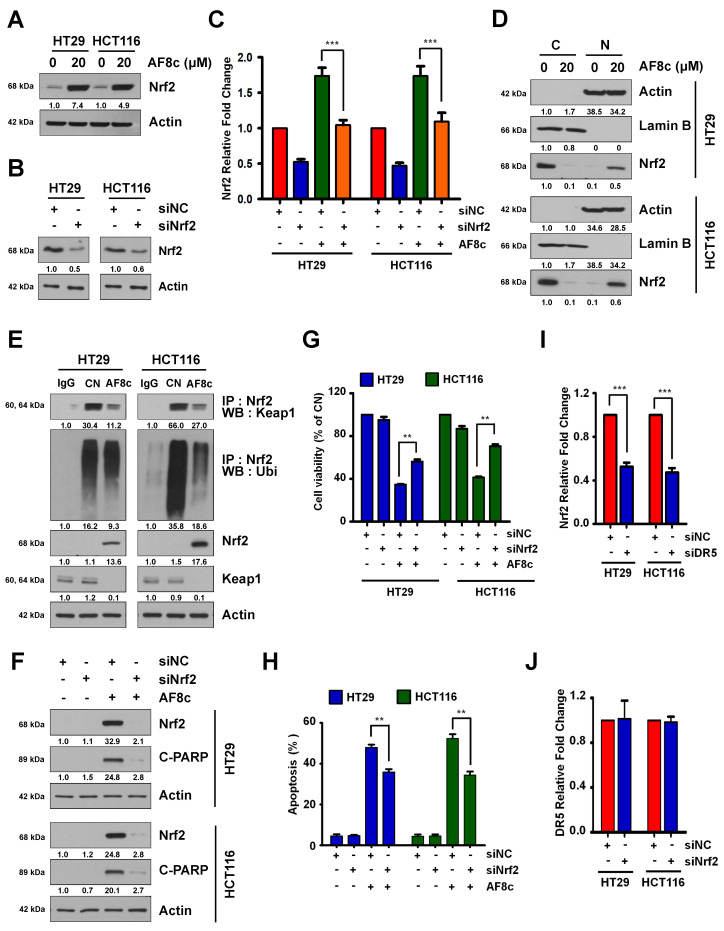
AF8c-induced apoptotic cell death is related to Nrf2 activation: (**A**) cells were incubated with 20 μM AF8c, and the Nrf2 protein levels were monitored by Western blotting; (**B**,**C**) HT29 (**left**) and HCT116 (**right**) cells were transfected with siNC or siNrf2 and then treated with 20 μM AF8c. The protein (**B**); and mRNA (**C**) expression levels of Nrf2 were subjected to qRT-PCR and Western blot; (**D**) AF8c-treated cells were fractionated, and Nrf2, Actin, and Lamin B were detected by immunoblotting: (**E**) after AF8c treatment, binding between Nrf2 and Keap1, and between Nrf2 and Ubiquitin was performed by immunoprecipitation; (**F**–**H**) after transfection with siNC or siNrf2, apoptosis resulting from AF8c treatment was analyzed by Western blotting (**F**); cell viability assay (**G**); and FACS analysis (**H**). **, *p* < 0.01. (**I**) Cells were transfected with siNC or siDR5, and the Nrf2 mRNA level was measured by qRT-PCR. ***, *p* < 0.001. (**J**) Cells were transfected with siNC or siNrf2, and DR5 mRNA levels were then confirmed by qRT-PCR.

Next, we hypothesized that AF8c-induced ROS overproduction could be regulated by the redox-sensitive transcription factor Nrf2. To test this, HT29 and HCT116 cells were treated with 20 μM AF8c, and the levels of Nrf2 protein were measured by immunoblotting assay. Treatment with AF8c markedly increased Nrf2 protein levels in both cell lines (Figure 4A). Consistent with this, Nrf2 knockdown inhibited the AF8c-induced increase in Nrf2 mRNA and protein levels (Figure 4B,C,F). The Nrf2 is normally ubiquitinated by Keap1, but when ROS is generated, Nrf2 enters the nucleus to regulate the antioxidant levels [31]. To verify this, we performed cytosolic and nuclear fractions as well as IP to examine the binding of Keap1 to Nrf2 and ubiquitination of Nrf2. As shown in Figure 4D,E, AF8c inhibited the binding of Nrf2 to Keap1 and promoted nuclear Nrf2 translocation. Furthermore, the apoptosis induced by AF8c was greatly attenuated by NRF2 silencing (Figure 4F–H). To determine whether Keap1, an E3 ligase of Nrf2, is involved in Nrf2 changes caused by AF8c, Keap1 was knocked down with siRNA, and then the cells were treated with AF8c. As a result, Nrf2 reduction by AF8c was further attenuated by siKeap1 (Figure S4A).

We further explored the AF8c-modulated relationship between DR5 and Nrf2 by transfecting HT29 and HCT116 cells with a DR5 siRNA and examining Nrf2 mRNA and protein levels. Knockdown of DR5 led to suppressed Nrf2 mRNA and protein levels (Figure 4I) and attenuated the partial reduction in the levels of Nrf2 mRNA induced by AF8c treatment (Figure S3A). Exposure to AF8c increased the levels of the Nrf2 target gene HO-1 and decreased the levels of Keap1, which degrades Nrf2. However, DR5 silencing decreased HO-1 and increased Keap1 levels induced by AF8c treatment (Figure S3B,C). In contrast, Nrf2 knockdown did not affect DR5 mRNA expression levels (Figure 4J).

Additionally, as p53 is known to regulate DR5 [32], we examined whether p53 is involved in the AF8c-mediated activation of DR5. The AF8c-induced increase in the levels of Nrf2, DR5, and apoptosis was diminished by p53 downregulation (Figure S3D, upper) or knockout (Figure S3D, lower). Collectively, our findings demonstrate that activation of p53-induced DR5 in AF8c-treated CRC cells leads to upregulation of the Nrf2 expression.

### 3.5. AF8c Enhances Apoptotic Cell Death in PDC Cells through Activation of DR5 and Nrf2 by Increased ER Stress

**Figure 5:**
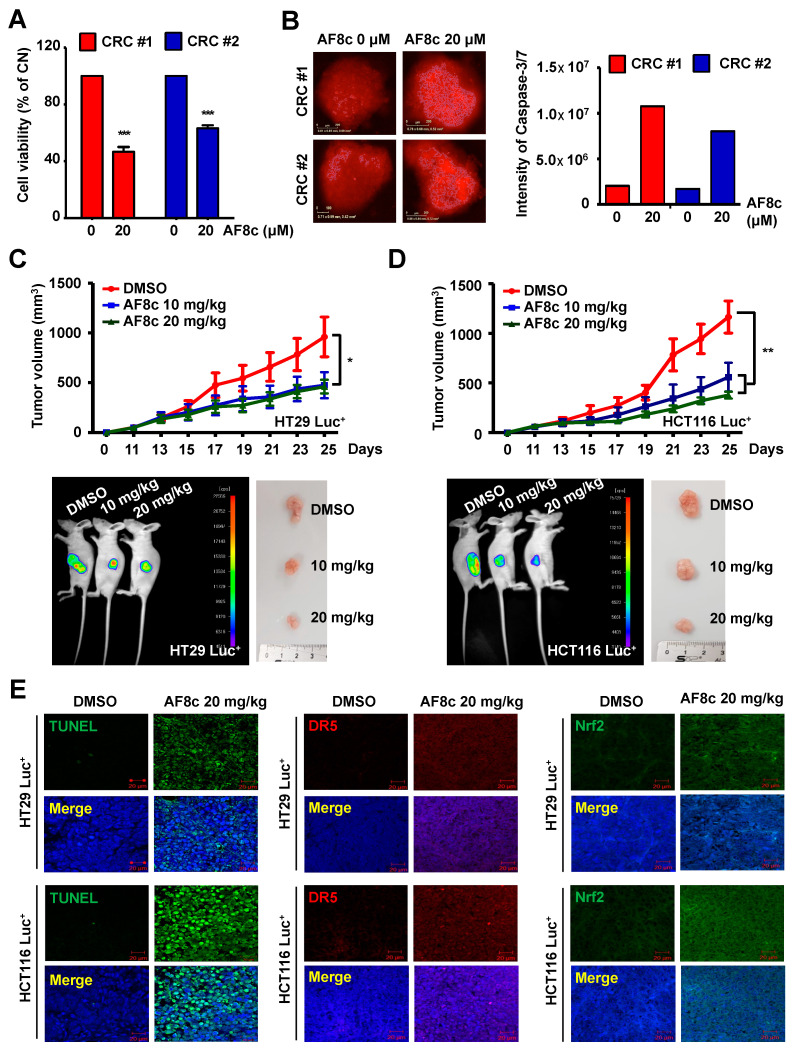
AF8c causes apoptosis by upregulating DR5, Nrf2, and CHOP expression in PDC cells and in vivo: (**A**) PDC cells were treated with AF8c for 24 h. Then, cell viability was measured by WST-1 assay. **, *p* < 0.01 and ***, *p* < 0.001; (**B**) the spheroid shapes were photographed with an optical microscope. After AF8c treatment, caspase 3/7 dye was added to the media and placed into an IncuCyte ZOOM for 3 days. Then, caspase 3/7 fluorescence intensity using either the IncuCyte Live-Cell (**left**) or ImageJ (right). Scale bar, 100 μm; (**C**,**D**) Subcutaneous injection of HT29 Luc^+^ (**C**) and HCT116 Luc^+^ (**D**) cells into the flanks of BALB/c nude mice. The upper panel indicates tumor volume in xenografts of nude mice while the lower-left panel illustrates the images of tumors obtained using a charge-coupled camera from the in vivo imaging system. The lower-right panel shows the tumor tissue isolated from xenograft nude mice photographed with a digital camera. *, *p* < 0.05 and **, *p* < 0.01. (**E**) Representative fluorescence images showing TUNEL assay results (**left**), Nrf2 (**middle**), and DR5 (right). The stained tissues were imaged using a confocal fluorescence microscope. Scale bar, 20 μm.

To confirm the cytotoxic effects of AF8c on CRC cells, we examined the effects of AF8c on patient-derived colorectal cancer (PDC) cells by treating two PDC cell lines (Figure S5A,B) with AF8c and assessing the cell viability. In both PDC lines, AF8c reduced cell viability (Figure 5A). Moreover, spheroids produced by PDC cells were loosened by treatment with AF8c. Exposure to AF8c markedly increased the fluorescence intensity of caspase 3/7 and decreased PDC cell viability (Figure 5B). To unveil whether levels of ER stress, DR5, and Nrf2 were elevated by AF8c treatment as observed for the CRC cells, PDC cells were incubated with AF8c. As shown in Figure S5C, the expression levels of cleaved PARP, DR5, Nrf2, and ER stress-related proteins were augmented, indicating that AF8c activates DR5, Nrf2, and ER stress response, leading to apoptosis in PDC cells.

### 3.6. AF8c Suppresses Tumor Growth In Vivo by Increasing DR5, Nrf2, and CHOP Expression and Subsequent Apoptosis

Based on our in vitro results, we confirmed the effects of AF8c in vivo using a xenograft mouse model. HT29 Luc^+^ and HCT116 Luc^+^ cells (1 × 10^7^ in 100 μL) were injected into BALB/c nude mice, and tumor size was monitored every day for 25 days. When the tumor volume reached 100 mm^3^, AF8c (10 and 20 mg/kg) was administered intraperitoneally. As shown in Figure 5C,D, tumor volume and bioluminescence intensities in AF8c-injected mice were significantly reduced compared to those in control mice. To examine whether the AF8c-induced suppression of tumor growth was related to apoptosis, we performed a TUNEL assay and immunofluorescence staining. The expression levels of DR5, Nrf2, and CHOP, as well as the number of TUNEL-positive cells, were greatly increased in AF8c-injected tumors compared to those in control tumors (Figure 5E and Figure S5D). Together, our results show that upregulation of DR5 and Nrf2 by AF8c enhanced AF8c-induced apoptosis by increasing ER stress not only in vitro but also in vivo.

## 4. Discussion

Off-target activity refers to the biological activity of a certain molecule on unintended biological targets. This off-target activity is usually associated with the observed side effects of this molecule. However, in many cases, the off-target activity of certain drugs was found to contribute to its overall therapeutic effect. Lapatinib, the FDA-approved EGFR/HER2 kinases inhibitor, which is currently used for metastatic breast cancer treatment, is an example. It was reported that lapatinib upregulates DR4/5 in CRCs, thus sensitizing these cells to TRAIL therapy. This off-target activity of lapatinib is believed to contribute to its overall anticancer activity and suggests that the combination between lapatinib and TRAIL or TRAIL agonists could be synergistic.

We have previously reported a series of lapatinib hybrids as inhibitors of EGFR/HER2 kinases. Of them, compound AF8c was chosen to further explore its mechanism of action in CRC cell lines and compare its activity with that of lapatinib. This is because it was able to inhibit the proliferation of HT29 and HCT116 CRC cells while sparing CCD-18CO normal epithelial primary colon cells. In addition, since the structural difference between AF8c and lapatinib is in their solvent-exposed moieties (in respect to their design rationale as kinase inhibitors), we believe that this moiety could contribute to the observed selective upregulation of DR5 expression by AF8c, which could provide a tool for understanding the impact of selective DR5 upregulation in cancer cells. However, this requires further experimentation to be confirmed. Interestingly, AF8c sensitized HT29 and HCT116 CRC cells to TRAIL in a similar manner to lapatinib. This could mean that AF8c might be beneficial in combination therapy with TRAIL or TRAIL agonists, yet further study might be needed to confirm this.

To date, only DR4 and DR5 among the TRAIL receptors have been implicated in apoptosis [33]. TRAIL binding to DR4 and DR5 induces their trimerization. This complex recruits Fas-associated protein with death domain (FADD) and pro-caspase 8, resulting in the formation of the death-inducing signaling complex (DISC). This leads to the activation of caspase 8 and ultimately results in apoptosis [34]. Our results showed that treatment of CRC cells with AF8c increased DR5 mRNA and protein levels and increased cleaved caspase 8 levels. As AF8c inhibits the proliferation of CRC cells by various mechanisms, silencing of DR5 decreased the levels of cleaved PARP, decreased apoptosis, and rescued the CRC cells partially.

Post-translational modification of proteins, protein folding, and protein transportation is carried out inside the Endoplasmic Reticulum (ER). Prolonged exposure to intracellular or extracellular insults disturbs the function of the ER, which is known as ER stress. Prolonged ER stress induces apoptosis via triggering large-scale intracellular cascades ultimately resulting in the initiation of CHOP transcription to prevent further accumulation of misfolded proteins via attenuation of the mRNA translation under ER stress [35]. CHOP is known to be one of the most crucial mediators of ER stress-induced apoptosis proteins, which also regulates DR5 expression [36,37]. The expression of DR5 is regulated by CHOP binding to the 5′-flanking region of the *DR5* gene [38], which could suggest that AF8c-induced DR5 activation in CRC cells may result from AF8c regulating CHOP binding to the *DR5* gene.

Under ER stress conditions, IRE1α is activated via autophosphorylation [35], ultimately leading to the dimerization and subsequent auto-phosphorylation of PERK (*p*-PERK) [39,40]. This leads to phosphorylation of eIF2α, which allows the translation of UPR-dependent genes such as ATF4 [39]. Consistent with this, we observed that AF8c treatment activated the UPR, as well as apoptosis. AF8c treatment leads to increasing the phosphorylation of eIF2α, IRE1α, PERK, and ATF4. In addition, AF8c treatment increased the levels of ATF6, GRP94, Bip, CHOP, and DR5. Collectively, these results suggest that the apoptotic effects observed in CRC cell lines are attributed to the ER stress, in addition to its kinase inhibition profile.

The p53 protein is known to modulate DR4 and DR5 [32]. Exposure to AF8c did not affect DR4 protein levels either in the p53 mutant (HT29273H) cells or p53 wild-type HCT116 cells, which we used in our experiments, whereas DR5 was upregulated. Sylvanie Surget et al. reported that p53 directly regulates DR5 gene expression, but not that of DR4, by increasing caspase-8 recruitment in myeloma [32], suggesting that AF8c selectively activates caspase-8 by increasing p53-mediated caspase-8 recruitment, thereby selectively inducing apoptosis. This could provide an insight into why AF8c induced the overexpression of DR5 but not DR4 in the tested CRC cells, which would require further research to be reported in a future report. Collectively, our results highlight that DR5 activation is significantly important for AF8c-induced apoptosis in human CRC cells.

It was reported that ROS production is linked to ER stress [41]. The mitochondrial electron transport chain is the primary site of ROS generation [42], and intracellular ROS levels are suppressed by antioxidant enzymes such as glutathione peroxidase, catalase, and SODs [43]. Our results show that mitochondrial superoxide is overproduced in CRC cells and that the levels of SOD1, SOD3, and catalase are reduced following AF8c treatment; this implies that AF8c may promote ROS generation, especially that of mitochondrial superoxide, by disrupting the mitochondrial electron transport chain and reducing the levels of antioxidant enzymes. However, this would require further experimentation, which shall be discussed in future reports.

Nrf2, a major sensor of oxidative stress, is involved in ROS homeostasis by regulating antioxidant defense systems [44]. The activity of NRF2 is controlled by Keap1, which was initially proposed to act by binding and tethering transcription factors in the cytoplasm [31]. Several oxidative stresses can disrupt the Nrf2-Keap1 complex. In addition, PERK and eIF2α can phosphorylate NRF2 leading to the dissociation of the NRF2-Keap1 complex [45,46,47]. This leads to the release of Nrf2 for translocation into the nucleus to induce transcription of its target genes *HO-1* and *KLF9* by binding to antioxidant-responsive element sequences [48,49]. Klf9 suppresses the expression of various antioxidant genes such as *TXNRD2* that are not direct Nrf2 targets, thus enhancing ROS production [49]. Our results show that AF8c attenuated Keap1 and Txnrd2 levels and increased the levels of Nrf2 and its target genes *HO-1* and *KLF9*. The modulated expression of antioxidant enzymes such as SODs, HO-1, Klf9, and Txnrd2 may contribute to AF8c-induced ROS accumulation.

To summarize, the lapatinib derivate AF8c induces CRC cell apoptosis in vitro and in vivo via various pathways. As a polypharmacological molecule, the AF8c-induced apoptosis is triggered, at least in part, by excessive mitochondrial ROS production and subsequent DR5-dependent Nrf2 activation. Those effects might be considered as an off-target activity of AF8c, which was originally designed as an ErbB family kinases inhibitor, similar to lapatinib. The results showed that the knockdown of either ErbB family members or DR5 and Nrf2 partially abolished the AF8c-induced cell death. This could mean that AF8c-induced cell death has several mechanisms, some of which are described in this report, and further studies are needed to understand other potential modes of action of AF8c. Collectively, our results suggest AF8c is a highly effective polypharmacological small molecule with an encouraging safety profile, both in vitro and in vivo, for further evaluation as a treatment of CRC.

## Data Availability

The data are available on request from the corresponding author.

## Supplementary Materials

The following are available online at https://www.mdpi.com/article/10.3390/cancers14133043/s1, Figure S1: Inhibition profile of AF8c against ErbB family of receptor tyrosine kinases and EGFR mutations., Figure S2: AF8c induces apoptosis better than lapatinib and synergizes with TRAIL., Figure S3: Activating DR5 induced by AF8c regulates the Nrf2/Keap1 pathway., Figure S4: Nrf2 increase by AF8c is regulated by Keap1., Figure S5: AF8c induces DR5 and Nrf2 activation through ER stress in PDC cells, leading to apoptosis. Figure S6: Original Western blot figures.
