# Supplementary material for: AF8c, a Multi-Kinase Inhibitor Induces Apoptosis by Activating DR5/Nrf2 via ROS in Colorectal Cancer Cells
Source: Cancers (Basel). 2022 Jun 21;14(13):3043. doi: 10.3390/cancers14133043 (PMC9264837; doi:10.3390/cancers14133043)
Supplement: Supplementary file 1 [file cancers-14-03043-s001.zip › cancers-1567970-supplementary.pdf]

AF8c, a Multi-Kinase Inhibitor Induces Apoptosis by Activating DR5/Nrf2 via ROS in Colorectal Cancer Cells

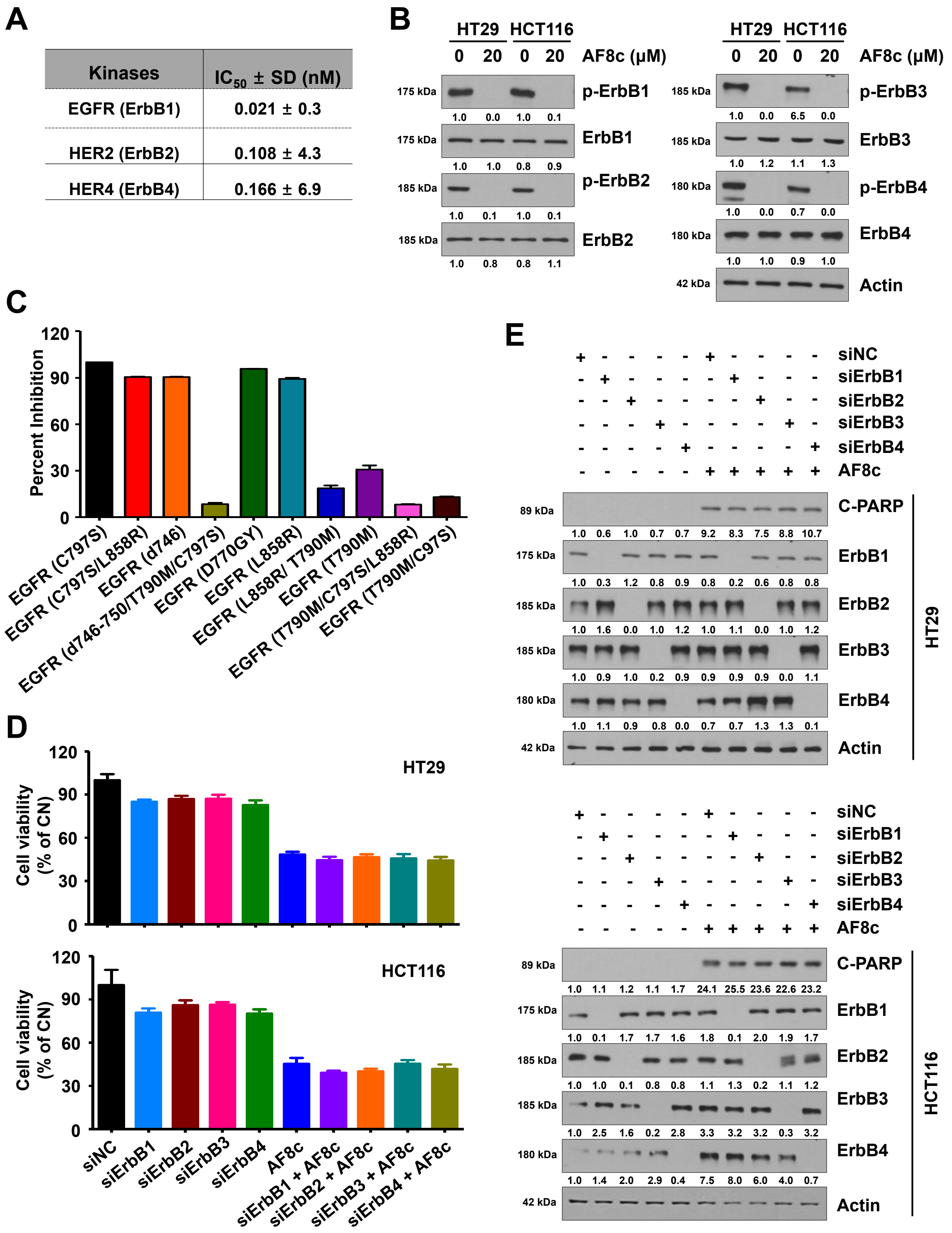

Kinases	IC_{50} ± SD (nM)
EGFR (ErbB1)	0.021 ± 0.3
HER2 (ErbB2)	0.108 ± 4.3
HER4 (ErbB4)	0.166 ± 6.9

Figure S1. Inhibition profile of AF8c against ErbB family of receptor tyrosine kinases and EGFR mutations. (**A**) $IC_{50} \pm$ SD values (nM) of AF8c against EGFR, HER2, HER4 kinases. (**B**) Cells were treated to AF8c for 24 h, and the expression levels of ErbB family members were determined by immunoblotting. (**C**) Percent inhibition values of AF8c against most common EGFR mutations. (**D–E**) HT29 (upper) and HCT116 (lower) cells were transfected with siRNAs. Then, cell viability (**D**) and cleaved PARP (**E**) were measured using the WST-1 assay and immunoblotting.

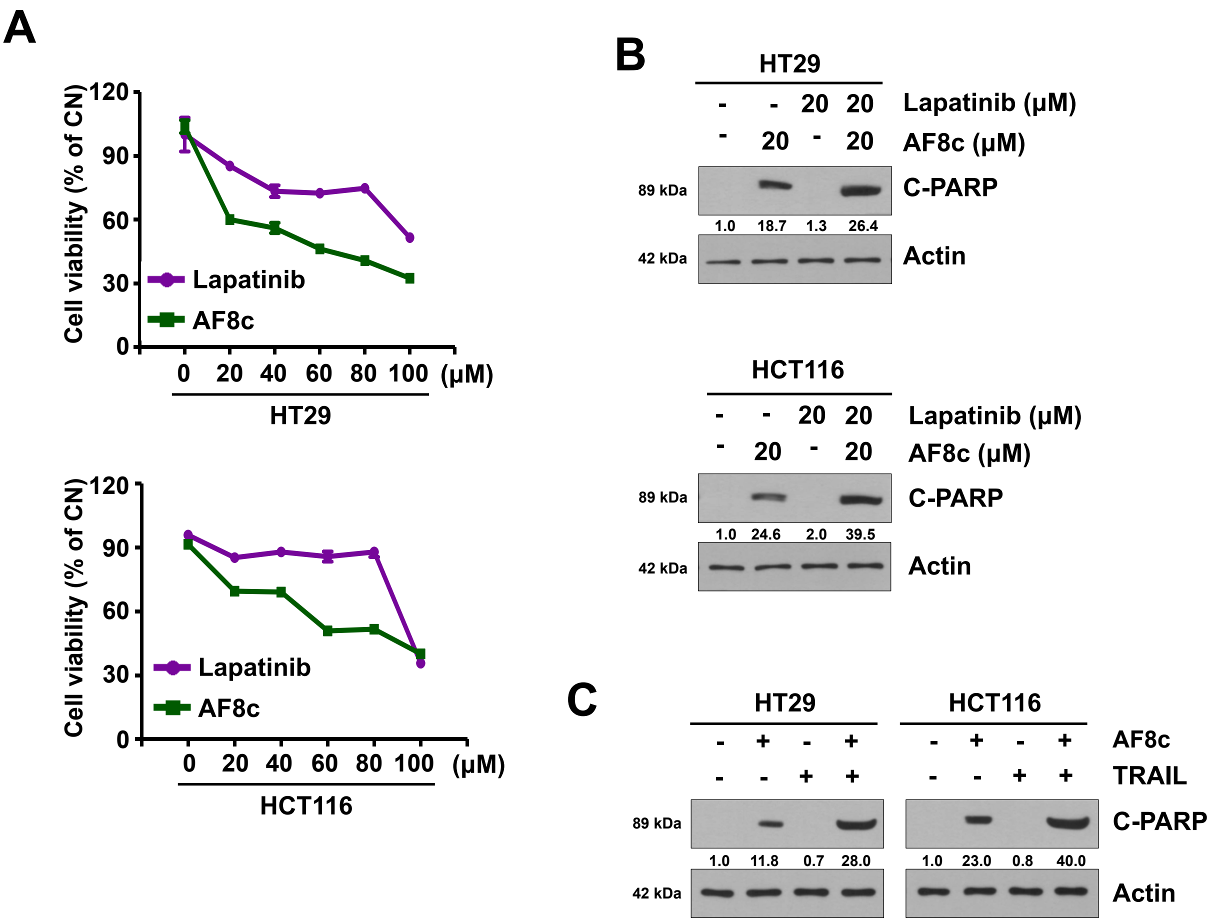

Figure S2. AF8c induces apoptosis better than lapatinib and synergizes with TRAIL. (**A–B**) HT29 (upper) and HCT116 (lower) cells were treated with 20 μM AF8c or lapatinib for 24 h. Then, cell viability (**A**) and Cleaved PARP (**B**) were assessed using the WST-1 assay and western blotting. (**C**) HT29 (left) and HCT116 (right) cells were treated with 20 μM AF8c and/or TRAIL for 24 h, then Cleaved PARP, Caspases 3, 9, and 8 were assessed using western blotting.

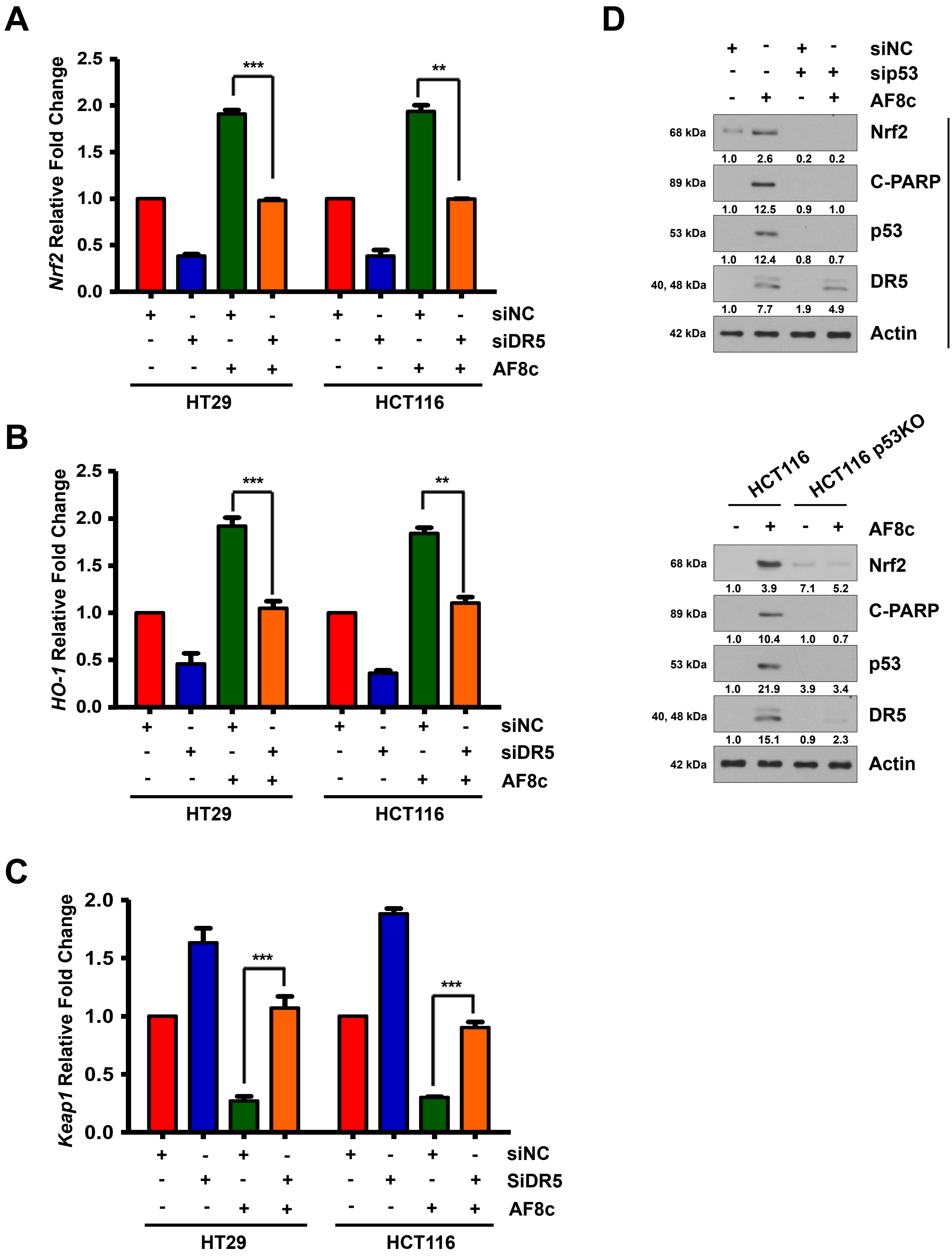

Figure S3. Activating DR5 induced by AF8c regulates the Nrf2/Keap1 pathway. (**A–C**) After transfection with siNC or siDR5, the expression of Nrf2 (**A**), Keap1 (**B**) and HO-1 (**C**) mRNA in response to AF8c were examined by qRT-PCR. **, $P < 0.05$ and ***, $P < 0.001$. (**D**) The cells incubated with 20 μM AF8c for 24 h, and subjected to immunoblotting in p53 knockdown (HT29, upper) or knockout (HCT116, lower) cells.

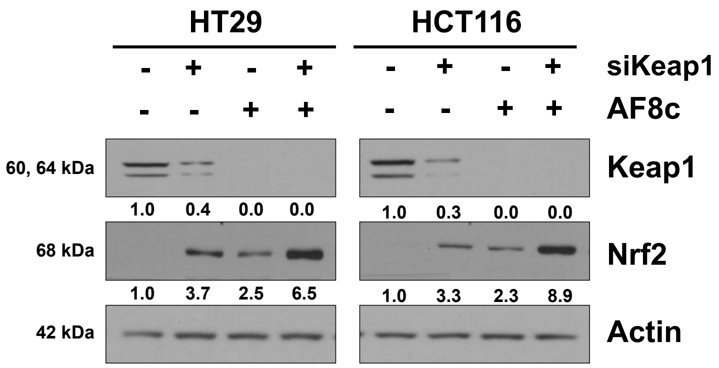

Figure S4. Nrf2 increase by AF8c is regulated by Keap1. HT29 (left) and HCT116 (right) cells were treated with 20 μM AF8c and/or siKeap1 and the levels of Keap1 and Nrf2 were measured by western blotting.

A

	Gender	Age	TMN stage (Pathology)	K/N RAS mutation (W or M)	Tumor EGFR (IHC)	Tumor c-Met (IHC)
CRC #1	M	61	T3N0MX	W	1	6
CRC #2	M	37	T4N0MX	W	2	3

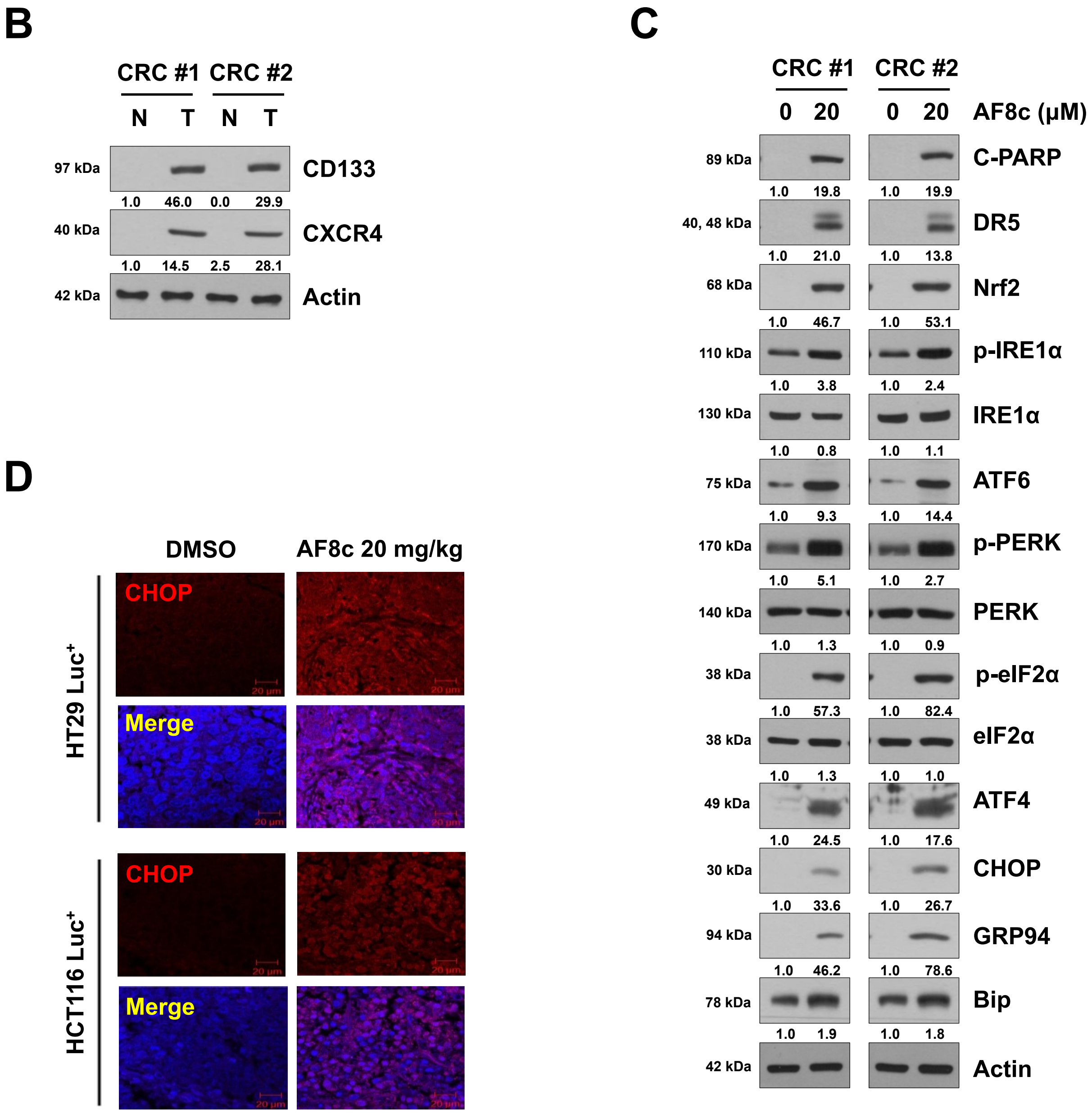

Figure S5. AF8c induces DR5 and Nrf2 activation through ER stress in PDC cells, leading to apoptosis. (**A**) Patient data and characteristics of the examined PDC cell lines. (**B**) Expression of CD133 and CXCR4 was examined by western blotting in cells derived from normal tissues and PDC cells. (**C**) AF8c-treated cells were detected for the apoptotic proteins, DR5, Nrf2, and ER stress related proteins by western blotting. (**D**) The tumor tissues isolated from xenografts were stained with CHOP and observed with confocal microscopy (Scale bars, 20 μm).

Fig. 1E

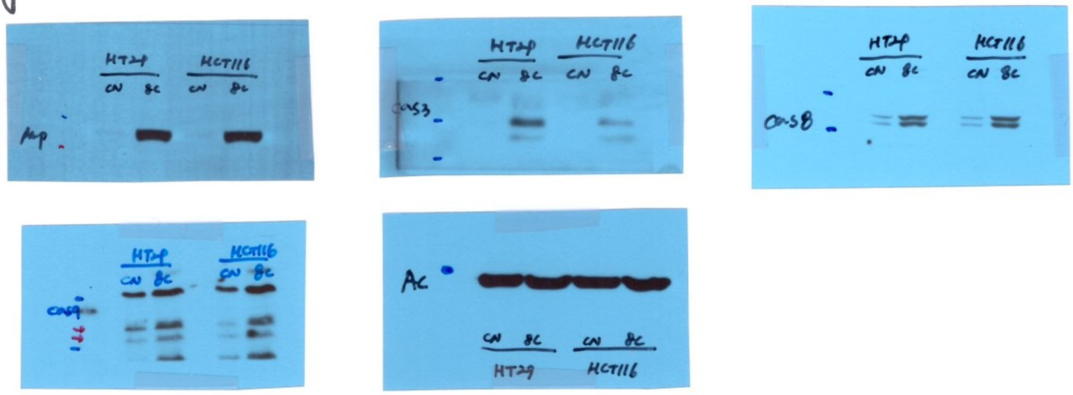

Fig. 2A

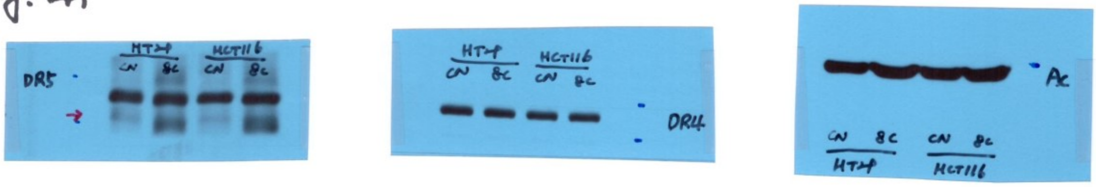

Fig. 2C

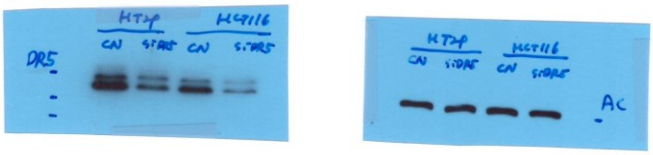

Fig. 2F

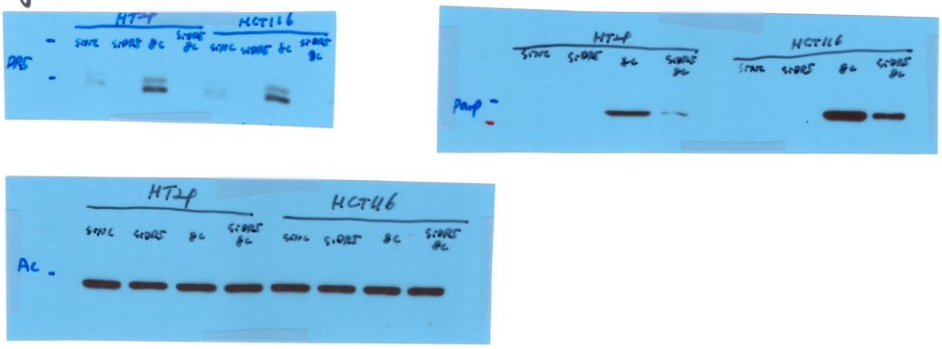

Fig. 3A

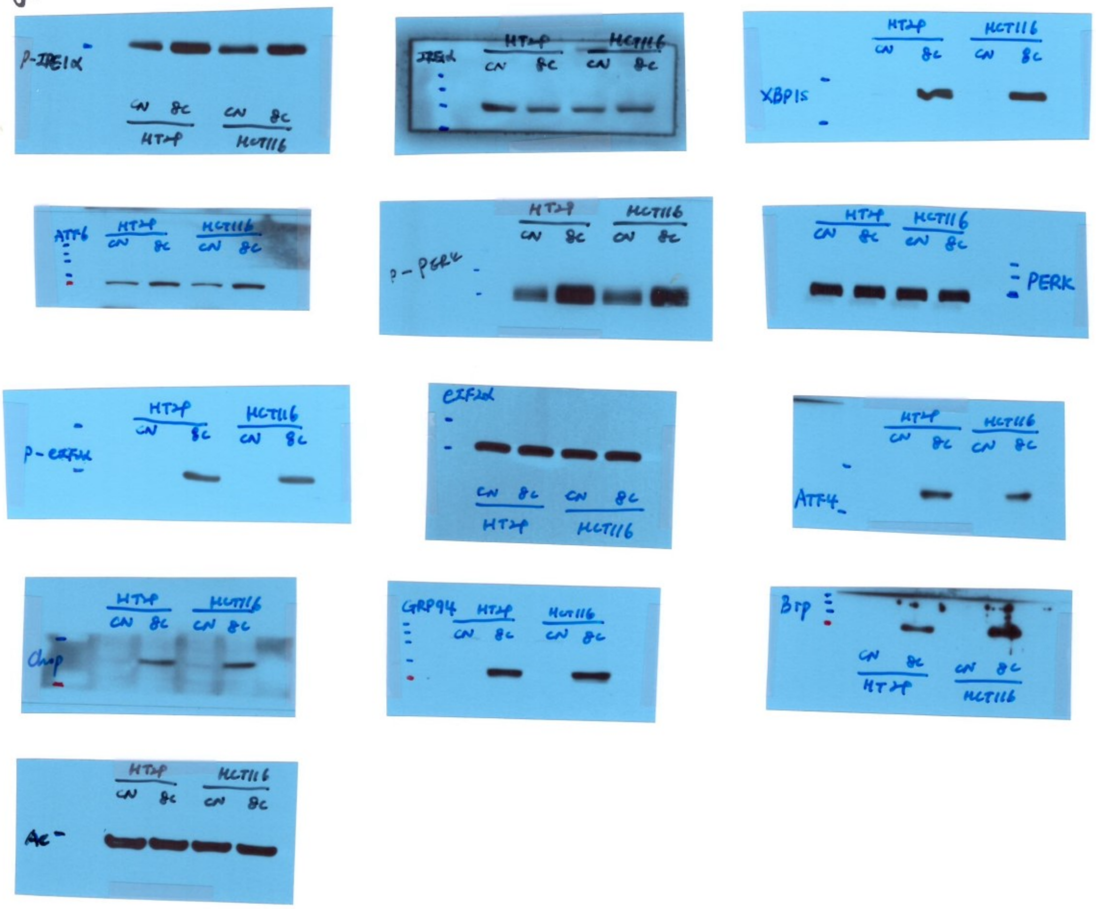
p-IRE1α
IRE1α
XBP1s
ATF6
p-PERK
PERK
p-eIF2α
eIF2α
ATF4
Chop
GRP94
Bip
Ac
HT29
HCT116
CN
8c

Fig. 3D

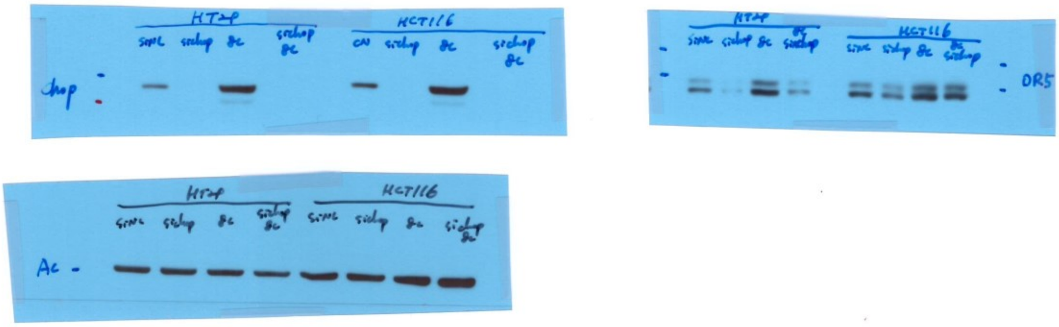
Chop
DR5
Ac
HT29
HCT116
siNC
sichop
8c
sichop 8c

Fig. 3E

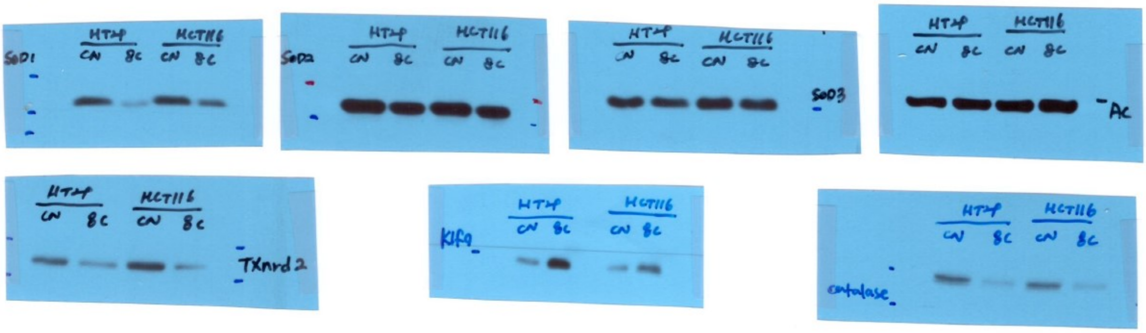
HT29
HCT116
CN
SC
SOD1
SOD2
SOD3
AC
TXnrd2
Klf9
catalase

Fig. 3H

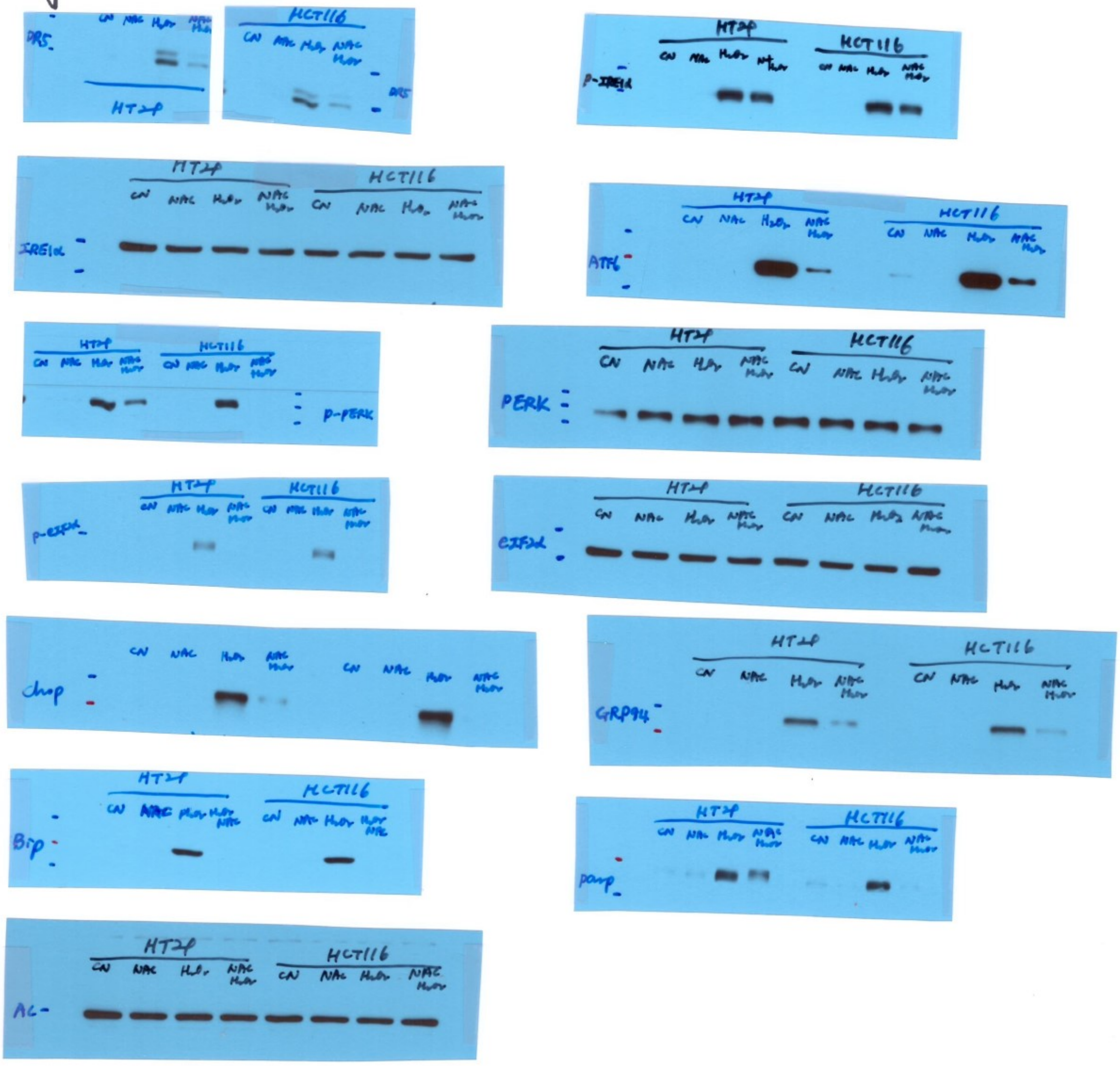
DR5
HT29
HCT116
CN
NAC
p-IRE1α
IRE1α
ATF6
p-PERK
PERK
p-eIF2α
eIF2α
chop
GRP94
Bip
AC

Fig. 4A

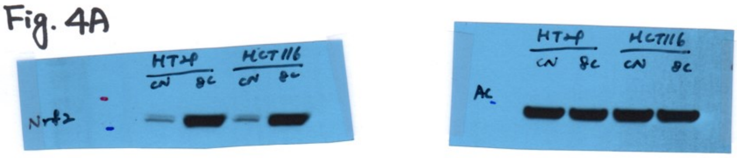

Fig. 4B

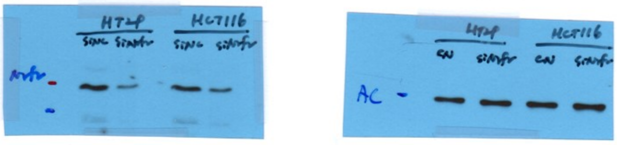

Fig. 4D

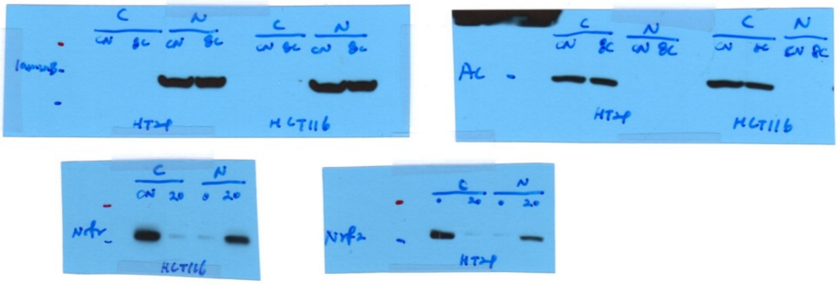

Fig. 4E

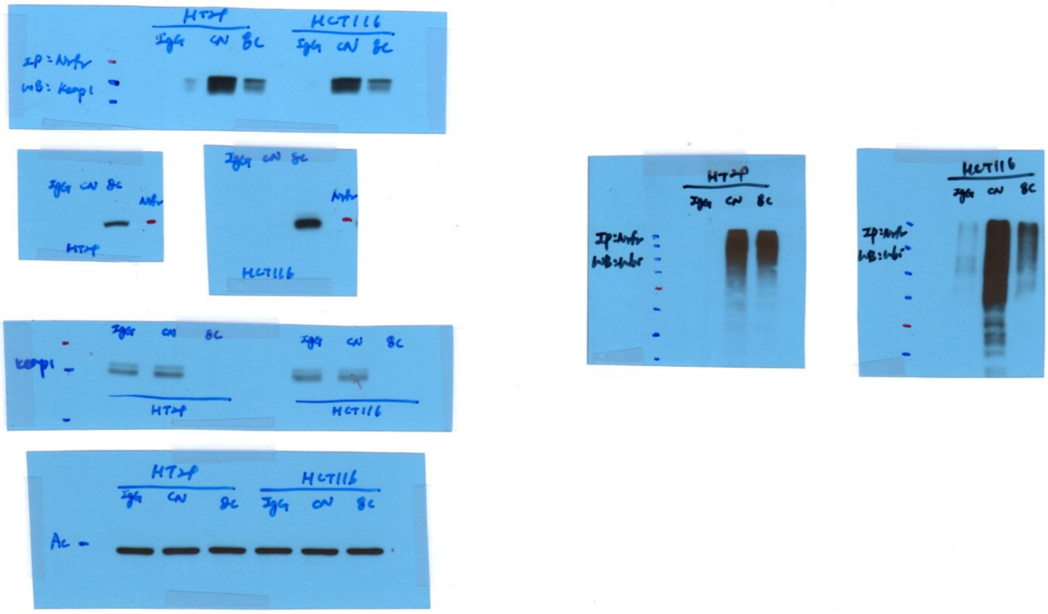

Fig. 4F

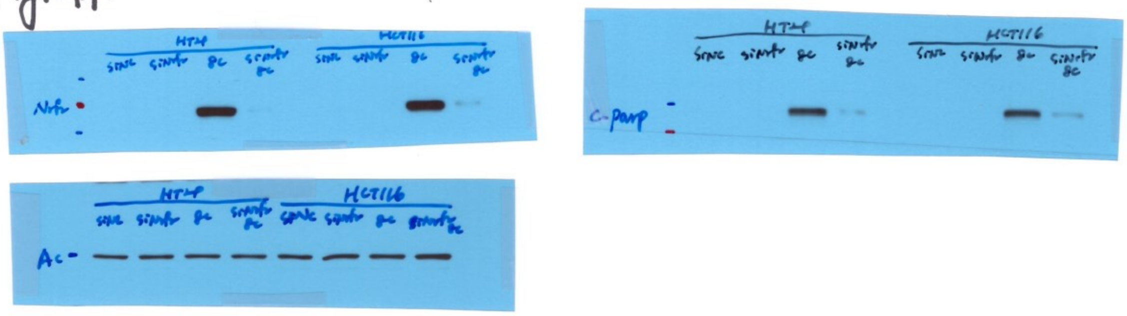

Fig. S1B

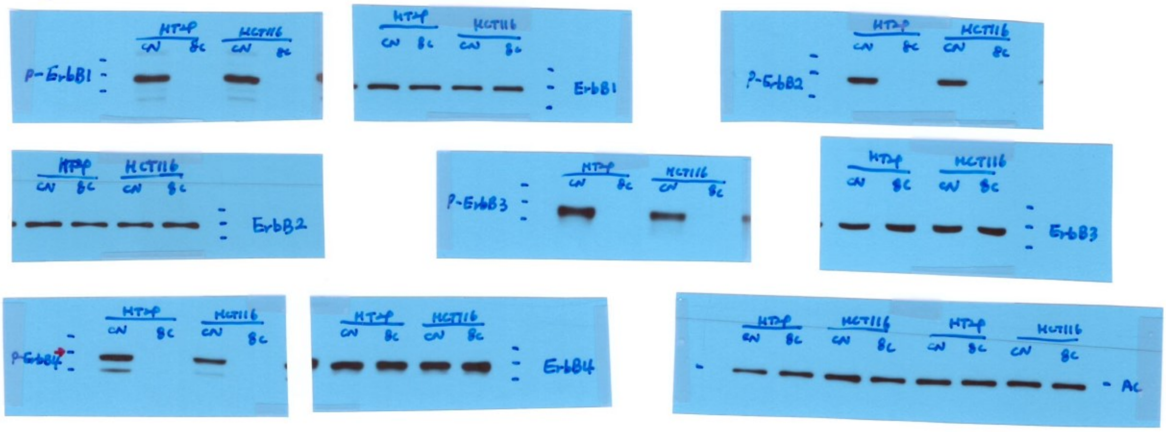

Fig. S1E

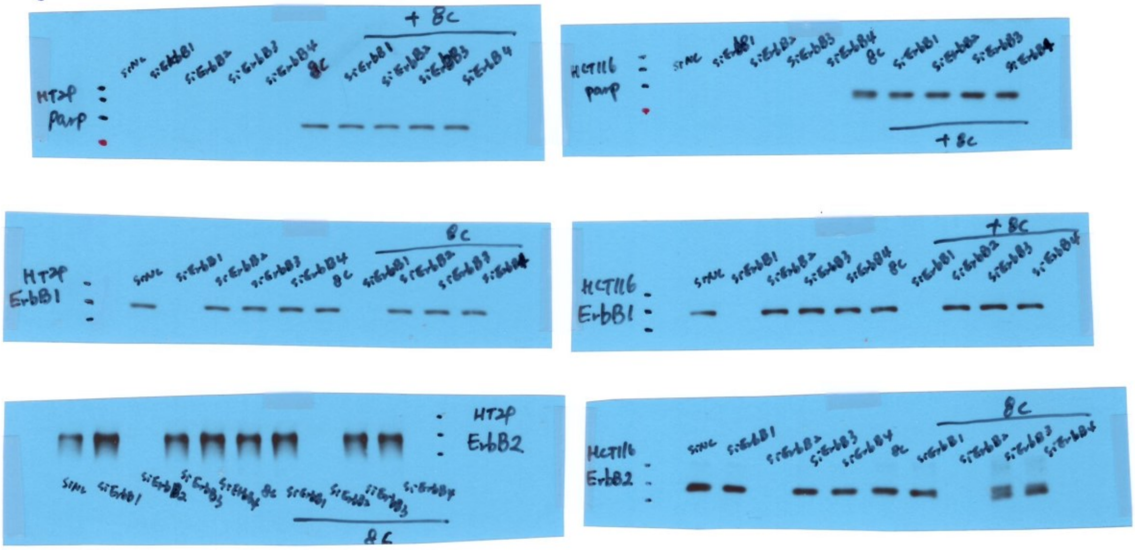

Fig. 4F

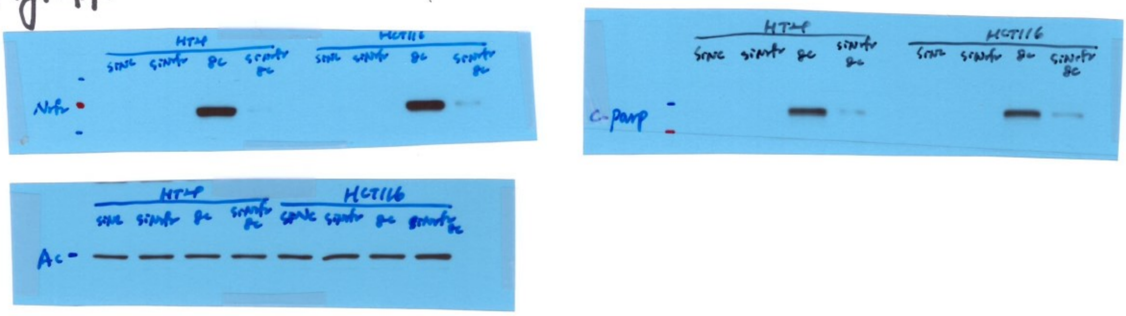

Fig. S1B

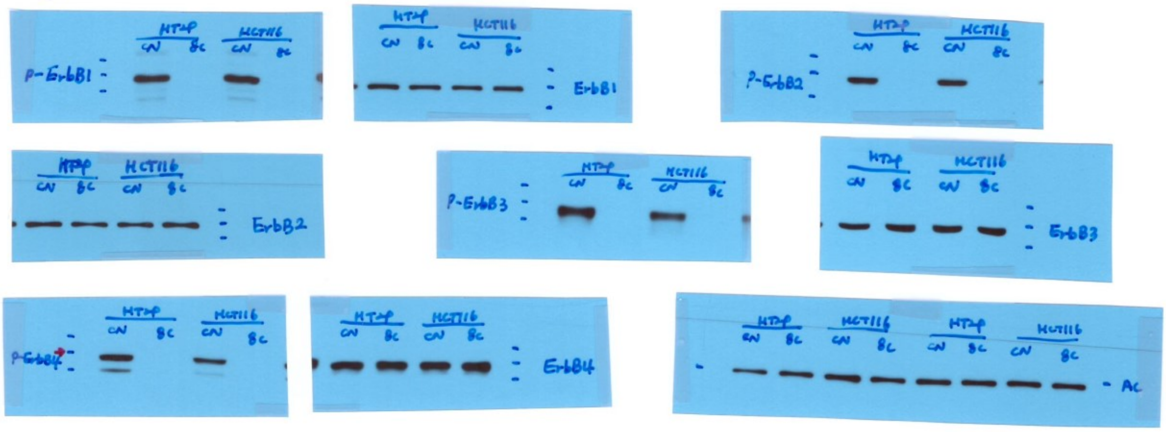

Fig. S1E

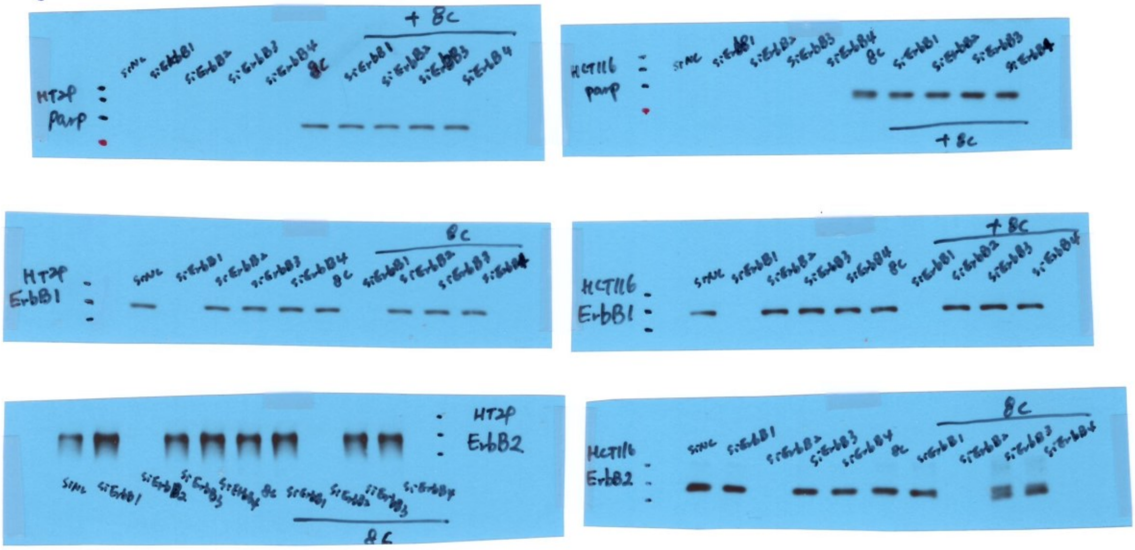

Fig. S1E

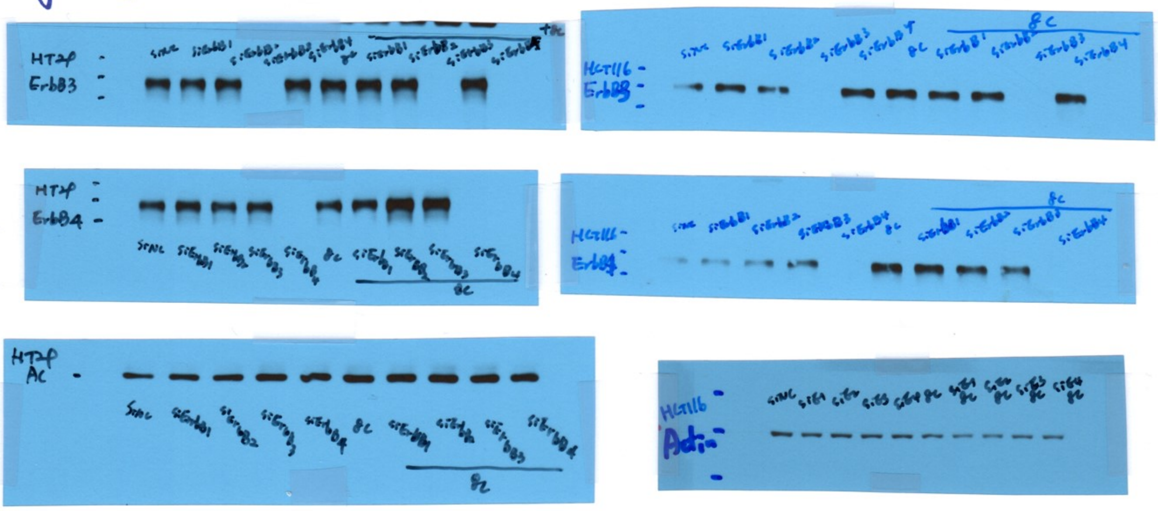

Fig. S2B

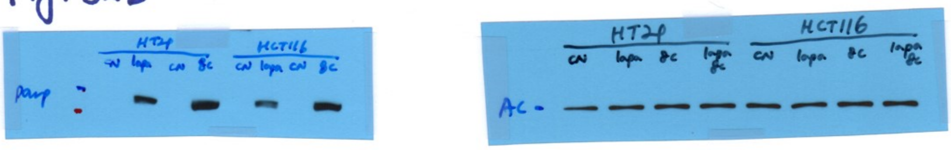

Fig. S2C

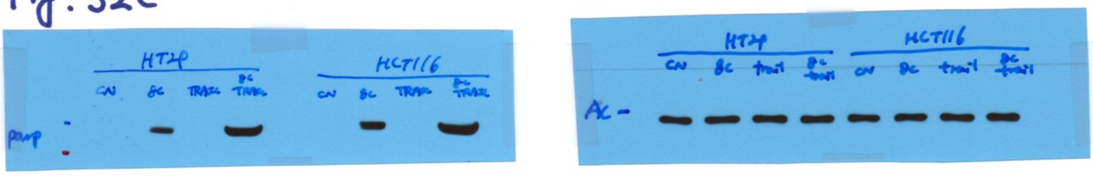

Fig. S3D

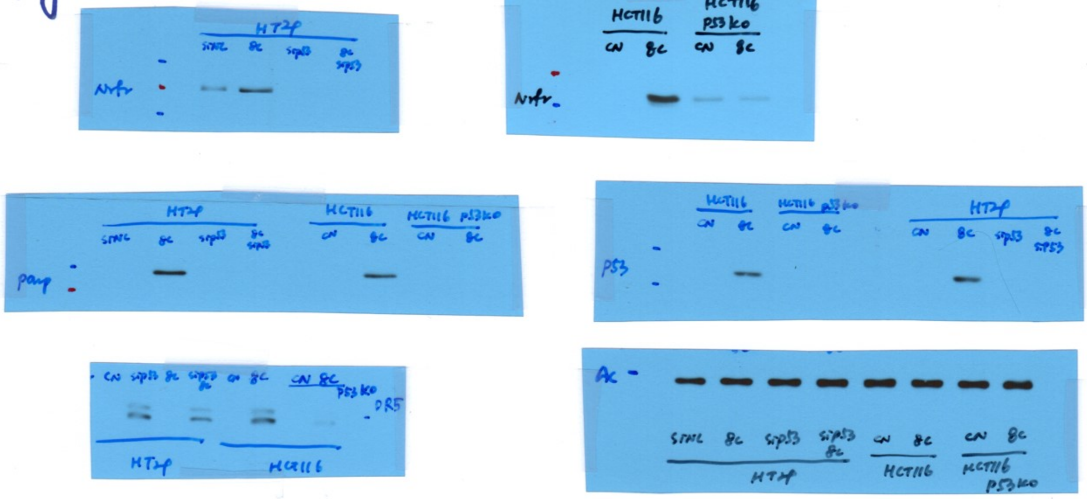

Fig. S4A

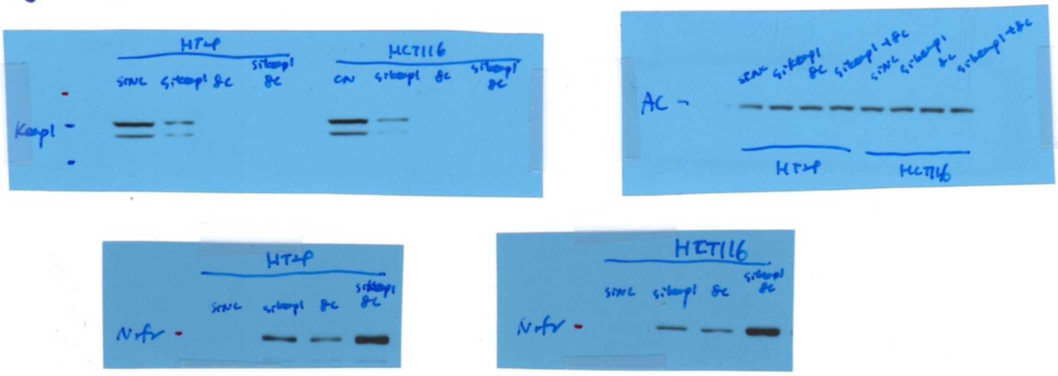

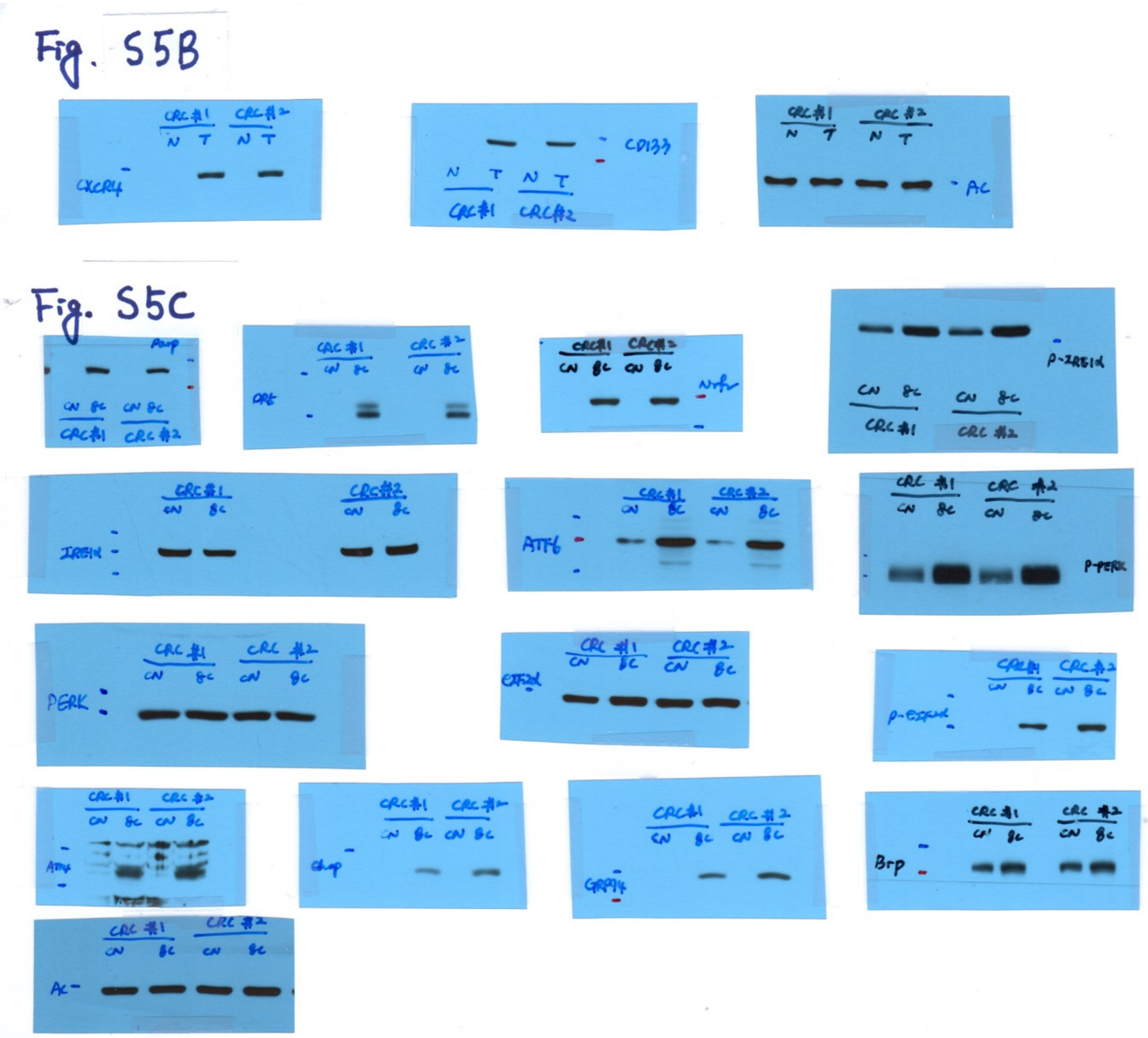

Figure S6. Original Western blot figures.
